# Kommunal verankerte Versorgungsstrukturen zur Förderung der Ernährungsgesundheit in den ersten 1000 Tagen – eine Analyse des aktuellen Status quo

**DOI:** 10.1007/s11553-022-00998-2

**Published:** 2022-11-28

**Authors:** Lena Schrameyer, Linda Wittler, Lisa Schmidt, Stefanie Wessely, Maria Flothkoetter, Stefanie Eiser, Katharina Reiss, Mechthild Paul, Nina Ferrari, Christine Joisten

**Affiliations:** 1 Institut für Bewegungs- und Neurowissenschaften, Abteilung für Bewegungs- und Gesundheitsförderung, Deutsche Sporthochschule Köln, Am Sportpark Müngersdorf 6, 50933 Köln, Deutschland; 2 Netzwerk Gesund ins Leben, Bundeszentrum für Ernährung (BZfE) in der Bundesanstalt für Landwirtschaft und Ernährung (BLE), Bonn, Deutschland; 3 Nationales Zentrum Frühe Hilfen, Bundeszentrale für gesundheitliche Aufklärung, Köln, Deutschland; 4 Kölner Zentrum für Prävention im Kindes- und Jugendalter, Herzzentrum der Uniklinik Köln, Köln, Deutschland

**Keywords:** Gesundheitsförderung, Kleinstkindalter, Ernährungsgesundheit, Verhältnisprävention, Verhaltensprävention, Health promotion, Early Childhood, Nutritional health, Community based prevention, Individual prevention

## Abstract

**Ziel:**

Die Ernährung in den ersten 1000 Tagen stellt einen relevanten Einflussfaktor für eine gesunde (Gewichts)entwicklung von Kindern dar. Verhältnispräventive, kommunalbasierte Ansätze scheinen am Erfolg versprechendsten in der Bekämpfung von (kindlichem) Übergewicht bzw. nicht übertragbaren Erkrankungen zu sein. Ziel dieser Arbeit war es, Strategien zum Gelingen der Ernährungsgesundheit in den ersten 1000 Tagen herauszuarbeiten.

**Methodik:**

Auf Basis einer internetbasierten Recherche möglicher kommunalbasierter Praxisprojekte wurden standardisierte, leitfadengestützte Interviews mit Expertinnen und Experten aus Wissenschaft und Praxis durchgeführt. Der Fokus lag auf der allgemeinen Einschätzung der Versorgungslage bzw. einem möglichen Handlungs- und Versorgungsbedarf sowie konkreten Handlungsempfehlungen unter besonderer Berücksichtigung von Schwangeren bzw. jungen Familien in belasteten Lebenslagen. 14 von 40 im Schneeballverfahren ausgewählten Personen nahmen teil.

**Ergebnisse:**

Alle Teilnehmenden schätzen die aktuelle primärpräventive Versorgung als nicht ausreichend ein. Als kritisch wurden von ihnen v. a. die fehlenden strukturellen und politischen Rahmenbedingen, insbesondere die häufig bestehenden zeitlich begrenzten Programme, gesehen. Dadurch seien Maßnahmen zur Förderung der Ernährungsgesundheit oder vergleichbare Interventionen nicht erfolgreich zu etablieren. Gefordert wurde daher eine deutlichere Unterstützung durch politische und andere relevante Stakeholder, z. B. Krankenversicherungen und Vernetzung mit bzw. Einbettung dieses Themenfeldes in den öffentlichen Gesundheitsdienst.

**Schlussfolgerung:**

Unsere Ergebnisse bestätigen aus Sicht von Expertinnen und Experten, dass die Förderung der Ernährungsgesundheit in den ersten 1000 Tagen sinnvoll und wichtig ist. Allerdings ist ein deutlicher Optimierungsbedarf hinsichtlich der Versorgungsstrukturen und der konkreten nachhaltigen Umsetzung von primärpräventiven Angeboten sowie niederschwelligen Zugängen für belastete Schwangere und Familien erkennbar.

**Zusatzmaterial online:**

Zusätzliche Informationen sind in der Online-Version dieses Artikels (10.1007/s11553-022-00998-2) enthalten.

## Abstract (en)

**Aim:**

Nutrition in the first 1000 days of life is a relevant influencing factor for healthy (weight) development of children. Community- and population-based preventive approaches seem to be the most promising way in counteracting overweight (in childhood) and noncommunicable diseases. The aim of this study was to analyze the health care situation in terms of promoting nutritional health in the first 1000 days.

**Methods:**

Based on an internet-based search of possible community-based practice projects, standardized, guideline-based interviews with experts from science and practice were conducted. The focus was on the general assessment of the health care situation or a possible need for action, as well as concrete recommendations, with special consideration of vulnerable groups. In all, 14 of 40 persons selected in a snowball procedure took part.

**Results:**

All experts rated the current health care situation in terms of nutrition as insufficient. They were critical of the lack of a structural and political framework, in particular the frequent time limits of a program. As a result, measures to promote nutritional health or comparable interventions could not be successfully established. Therefore, clearer support by political and other relevant stakeholders, e.g., health insurances, and networking with or embedding of this topic area in public health services was demanded.

**Conclusion:**

From the experts’ point of view, our results confirm that the promotion of nutritional health in the first 1000 days is meaningful and important. However, there is a clear need to optimize care structures and the concrete, sustainable implementation of primary prevention services as well as low-threshold access for pregnant women and families in precarious living situations.

## Einleitung

Der Lebensabschnitt zwischen der Empfängnis und dem 2. Geburtstag eines Kindes, die ersten 1000 Tage, spielen eine zentrale Rolle in der kindlichen Entwicklung [23]. Diese Phase ist gekennzeichnet durch ein schnelles Wachstum und eine enorme neuronale Entwicklung und damit auch einen hohen Nährstoffbedarf bzw. starke Empfindlichkeit gegenüber Programmierungseffekten. So nehmen nach der „Developmental origins of health and diseases“-Hypothese (DoHaD) eine Reihe von Umweltbedingungen während und nach der Schwangerschaft Einfluss auf die Programmierung der metabolischen Gesundheit des werdenden Organismus [15]. Dadurch scheint der Phänotyp der Nachkommen und das Risiko im späteren Leben an einer nicht übertragbaren Erkrankung wie Übergewicht zu erkranken, beeinflusst zu werden [14]. Dazu zählen neben der genetischen Determination auch Gen-Umwelt-Interaktionen, u. a. durch den elterlichen Lebensstil, insbesondere einer ausreichenden und qualitativ hochwertigen Versorgung mit Kalorien und Nährstoffen [14]. Umgekehrt kann eine unausgewogene Ernährung, v. a. eine Überernährung zur Entstehung von Übergewicht, Herz-Kreislauf-, Stoffwechsel- und endokrinen Erkrankungen führen [22]. Aus diesen Gründen scheinen im Kontext der sog. metabolischen Gesundheit sowohl die Ernährungsgewohnheiten der Mutter während der Schwangerschaft als auch die Wahl der verabreichten Nahrung an das Kind nach der Geburt eine Rolle zu spielen [24]. Ein erhöhtes Körpergewicht der Mutter während der Schwangerschaft sowie die Wahl einer eher proteinreichen Nahrung nach der Geburt sind – neben anderen Lebensstilfaktoren – mit einem erhöhten Risiko, im Laufe der nächsten Lebensjahre eine Adipositas bis hin zum metabolischen Syndrom zu entwickeln, assoziiert. Außerdem kommt es in der frühen Kindheit zur Entwicklung von Lebensmittelpräferenzen und einem Essverhalten, das zusätzlich durch die elterlichen Fütterungspraktiken und Rollenmodelle sowie den Bildungsgrad bzw. sozioökonomischen Status beeinflusst wird. Diese Vorlieben und Essgewohnheiten können sich bis ins spätere Leben fortsetzen und ihrerseits wiederum Einfluss auf den jeweiligen Gesundheitsstatus nehmen [35].

Zu möglichen Gruppen, bei denen ein besonderer Förderbedarf hinsichtlich eines gesunden Lebensstils besteht, werden Kinder aus Familien mit Migrationshintergrund, mit niedrigem sozioökonomischem Status gezählt bzw. wenn psychische Erkrankungen bei einem Elternteil vorliegen [38]. Bislang gibt es allerdings keinen Königsweg bzgl. entsprechender Maßnahmen [31]. Die höchste Reichweite weisen auf Basis der aktuellen Datenlage kommunal basierte Ansätze auf [3]. Ein Beispiel sind Präventionsketten bzw. integrierte Gesundheitsstrategien, die auf kommunaler Ebene einen Rahmen für verschiedenste Unterstützungsangebote öffentlicher und privater Träger und Akteurinnen und Akteure schaffen und Synergien nutzen sollen. Darüber hinaus sollen sie dazu beitragen, die jeweiligen Unterstützungsangebote über Altersgruppen und Lebensphasen hinaus aufeinander abzustimmen [29]. Damit sollen individuelle und zielgruppengerechte Maßnahmen vor Ort umgesetzt und mit bestehenden Angeboten und Strukturen vernetzt werden.

In der Realität finden sich bislang nur vereinzelt Erfolg versprechende integrierte Ansätze, wie z. B. „Café Kinderwagen“, „Begrüßungsbesuche bei Familien mit Neugeborenen“, „BEAGLE“, „HAG“, „Mo.Ki unter 3 frühes Fördern von Anfang an“. Innerhalb dieser Projekte ist es besonders gelungen, Eltern und Kinder über mehrere Lebensphasen durch vernetzte und zusammenarbeitende Fachkräfte und Akteurinnen und Akteure zu unterstützen.

Meist gibt es eher Einzelprojekte wie Beratungsgespräche und Schulungen für Eltern und Multiplikatorinnen und Multiplikatoren, Fachgespräche und -konferenzen über die Gestaltung einer gesunden Ernährung sowie die Etablierung konkreter Anlaufstellen in Form von Elterncafés oder Familienbüros. Um aber eine Trendwende hinsichtlich der Ernährungsgesundheit insbesondere in dieser frühen Phase erreichen zu können, empfiehlt sich eine flächendeckende und systematische Versorgung. Zu diesem Zweck wurde im Rahmen des hier vorgestellten Projekts die aktuelle Situation dargestellt und deren Beurteilung durch Expertinnen und Experten vorgenommen. Damit sollte die Grundlage für die Entwicklung möglicher Strategien zur Förderung der Ernährungsgesundheit in den ersten 1000 Tagen bzw. elterlichen Ernährungskompetenz und Handlungsempfehlungen für das Gelingen primärpräventiver Maßnahmen gelegt werden.

## Methodik

Die Analyse erfolgte im Rahmen des Projekts „Förderung der Ernährungsgesundheit in kommunalen Kleinstkindsettings“ unter Koordination des Netzwerks Gesund ins Leben mit den Kooperationspartnerinnen und Kooperationspartnern Nationales Zentrum Frühe Hilfen und dem Verband der Privaten Krankenversicherung e. V. (Steuerungsgruppe).

### Studiendesign

Als Grundlage für die Befragung erfolgte zunächst zwischen Mai und Dezember 2020 eine internetbasierte Recherche zur aktuellen Versorgungslage bzgl. nationaler (kommunal verankerter) Praxisprojekte zur Förderung der Ernährungsgesundheit im Kleinstkindalter in Deutschland. Da eine einheitliche Definition des Begriffs „Ernährungsgesundheit“ nicht existiert, erfolgte die Recherche explorativ nach Suchbegriffen bzw. möglichen Kombinationen, die mit einer gesunden bzw. ausgewogenen Ernährung in Verbindung gebracht werden: Ernährung, Präventionsprogramme, Empfehlungen, Kleinstkinder, Säuglinge, Schwangerschaft, Beikost Produkte Kleinkinder Ernährung Studien; Handlungsempfehlung Ernährung Schwangerschaft; Ernährung Säuglingsalter; Präventionsprogramme Ernährung Kleinkinder; Präventionsprogramme und Interventionsprogramme zur Ernährung von Kindern bis 2 Jahre; vegetarische Ernährung Kleinstkinder; Präventionsprogramme Kindergarten; Präventionsprogramme Säuglingsernährung; vegane Ernährung Säuglinge; Empfehlungen Stillen. Die Begriffe wurden in die deutschsprachigen elektronischen Datenbanken Google Scholar und Google eingegeben, um die vorhandene Literatur, Präventionsprogramme und Informationsmaterialien zur Gestaltung von Kleinstkind-Settings zur Förderung der Ernährungsgesundheit von Kleinstkindern (0 bis 2 Jahre), inklusive Ernährung in der Schwangerschaft, zu detektieren. Auch in dieser Recherche wurden nur Materialien berücksichtigt, deren Inhalt der Zielgruppe (Schwangere und Kleinstkinder bis 2 Jahre) entsprach und deren Schwerpunkt Ernährung war. Integriert wurden nur die Treffer, die sich auf gesunde, sich „normal“ entwickelnde Kleinstkinder sowie gesunde Schwangere bezogen. Zusätzlich wurden institutionelle Datenbanken identifiziert wie die des Robert Koch-Instituts, des Max Rubner-Instituts, des Bundesgesundheitsministeriums, des Kompetenzzentrum für Ernährung, der Deutschen Gesellschaft für Ernährung, des Forschungsdepartment Kinderernährung, der Bundeszentrale für gesundheitliche Aufklärung, des Kooperationsverbunds Gesundheitliche Chancengleichheit, des Netzwerks Gesund ins Leben und IN FORM und der Plattform für Ernährung und Bewegung und ebenfalls nach den oben genannten Begriffen durchsucht. Die jeweiligen Treffer wurden in Form einer spezifischen Recherche nochmals auf Stimmigkeit der Inhalte und Zielgruppen überprüft. Zudem wurden per Handsuche in den einzelnen Literaturverzeichnissen weitere Autorinnen und Autoren identifiziert und deren nationale und internationale Interventionen bzw. Good-practice-Modelle sowie Forschungsvorhaben (inklusive E-Health) zur Ernährungsgesundheit recherchiert. Die Informationen wurden auf Basis eines Rasters aus den Internetseiten bzw. – wenn vorhanden – Dokumenten extrahiert und wie folgt dargestellt: Zunächst wurde eine Kurzinformation über das entsprechende Projekt erstellt und anschließend jedes Praxisprojekt in eine Tabelle mit folgender Clusterung eingetragen: Laufzeit, Träger:in/Institution; Themengebiet; für Zielgruppe relevante Informationen; Ergebnisse; Sonstiges; Quelle. Im Feld Sonstiges wurde zusätzlich die eventuelle Berücksichtigung der entwicklungspsychologischen Aspekte innerhalb des jeweiligen Programms aufgeführt. Im Anhang 1 findet sich die Zusammenstellung der kommunal verankerten Projekte (s. Anhang Tab. 1S); aufgrund des großen Umfangs werden die übrigen Praxisprojekte hier nicht gezeigt. Zusätzlich wurde eine weitere Tabelle angelegt, in die die aussortierten Projekte aufgenommen wurden. Auch hier erfolgte die Clusterung nach: Titel; Träger; Grund für Ausschluss (s. Anhang Tab. S2; dargestellt wurden ebenfalls nur die für dieses Manuskript relevanten Bezüge).

Auf Basis dieser Recherche wurden die Leitfragen für die Expertinnen- und Experteninterviews formuliert, um mögliche Wissenslücken u. a. hinsichtlich neuer Forschungsergebnisse wie der Bedeutung der frühen Programmierung zu schließen und Handlungsempfehlungen zu generieren (Tab. 1).

**Tab. 1:** Übersicht über die Leitfragen

*Einleitende Fragen zur Person*	
1)	Angaben zur Person und Qualifikation
2)	An welchen Programmen und/oder Studien zum Thema Ernährung oder Gesundheitsförderung von Kleinstkindern und Ernährung von Schwangeren haben Sie mitgearbeitet und in welchen Lebenswelten (s. § 20 Absatz 4 Nummer 2)? a) soziales Wohnen bzw. Wohnumfeld b) soziales System Lernen bzw. Lernumfeld/Bildungsstätten c) soziales System medizinische und pflegerische Versorgung d) soziales System Freizeitgestaltung einschließlich Sport e) Individualangebote
*Einschätzung der allgemeinen Situation/Handlungs- und Versorgungsbedarf im Kontext der fokussierten Zielgruppen*	
3)	Für wie wichtig erachten Sie generell Präventionsprogramme mit dem Fokus Ernährung und warum? Nennen Sie die aus Ihrer Sicht wichtigsten drei Gründe
4)	Wie bewerten Sie die aktuelle primärpräventive Versorgungssituation a) in Kommunen? b) allgemein?
*Fragen zur Ausgestaltung von Programmen/Ideen für Versorgungsziele/-strategien*	
5)	Wie sollten Präventionsprogramme Ihrer Meinung nach aufgebaut sein? Im a) sozialen Wohnen bzw. Wohnumfeld b) sozialen System Lernen bzw. Lernumfeld/Bildungsstätten c) sozialen System medizinische und pflegerische Versorgung d) sozialen System Freizeitgestaltung einschließlich Sport e) Individualangebote
6)	Welche Schwerpunkte sind für solche Präventionsprogramme Ihrer Meinung nach besonders wichtig? 6.1) allgemeine Nennung und Begründung 6.2) spezifisch: Welches der Themengebiete Ernährungsweise, Ernährungsverhalten, Ernährungswissen, Ernährungseinstellung und Ernährungskompetenz ist Ihrer Meinung nach am wichtigsten und warum? 6.3) Wie können/sollten diese Themengebiete ausgestaltet werden? 6.4) Wie werden bzw. wie können entwicklungspsychologische Aspekte integriert werden?
7)	Was sind Ihrer Meinung nach Erfolgsfaktoren für das Gelingen präventiver Angebote (mit dem Schwerpunkt Ernährung) a) auf Basis Ihrer Expertise b) allgemeine Ergänzungen zu weiteren Zielgruppen und Lebenswelten
8)	Und was sind Ihrer Meinung nach Aspekte, die den Erfolg solcher präventiven Angebote erschweren oder verhindern? a) auf Basis Ihrer Expertise b) allgemeine Ergänzungen zu weiteren Zielgruppen und Lebenswelten
9)	Wie sollte/könnte man die Nachhaltigkeit im Sinne von Verstetigung verbessern? a) auf Basis Ihrer Expertise b) allgemeine Ergänzungen zu weiteren Zielgruppen und Lebenswelten
10)	Welche (kommunalen, infrastrukturellen) Rahmenbedingungen müssen gegeben sein, damit ein sozial gerechtes Präventions- und Versorgungsprogramm erfolgreich durchgeführt werden kann (z. B. politisch, inhaltlich etc.)? a) auf Basis Ihrer Expertise b) allgemeine Ergänzungen zu weiteren Zielgruppen und Lebenswelten
11)	Welche Partnerinnen und Partner werden für eine erfolgreiche und nachhaltige Umsetzung benötigt? a) auf Basis Ihrer Expertise? b) allgemeine Ergänzungen zu weiteren Zielgruppen und Lebenswelten
*Fragen zur Erreichbarkeit vulnerabler Gruppen*	
12)	Welche Gruppen halten Sie für schwer erreichbar? Welche Bedarfe und Barrieren sehen Sie und welche Erfahrungen mit Gesundheitsförderung in vulnerablen Gruppen haben Sie?
13)	Welche Zugangswege können/sollten zur Erreichung dieser Gruppen genutzt werden? Über wen sollte die Ansprache erfolgen? a) auf Basis Ihrer Expertise b) allgemeine Ergänzungen zu weiteren Zielgruppen und Lebenswelten
14)	An welchen Lebenswelten kann/sollte man anknüpfen (z. B. Geburtshäuser)? a) auf Basis Ihrer Expertise b) allgemeine Ergänzungen zu weiteren Zielgruppen und Lebenswelten
15)	Welche inhaltlichen Schwerpunkte sollte man bei der Kommunikation setzen? a) auf Basis Ihrer Expertise b) allgemeine Ergänzungen zu weiteren Zielgruppen und Lebenswelten
16)	Welche Rolle spielen dabei entwicklungspsychologische Aspekte? a) auf Basis Ihrer Expertise b) allgemeine Ergänzungen zu weiteren Zielgruppen und Lebenswelten
17)	Welche Möglichkeiten der Vermittlung schlagen Sie vor (u. a. Kommunikationsmedien)? a) auf Basis Ihrer Expertise? b) allgemeine Ergänzungen zu weiteren Zielgruppen und Lebenswelten
18)	Gibt es Ihrer Einschätzung nach Themen oder Aspekte, bei denen man sehr vorsichtig sein sollte bei der Ansprache, um nicht aus Versehen unerwünschte Effekte oder Ablehnung bei den Zielgruppen auszulösen? a) auf Basis Ihrer Expertise? b) allgemeine Ergänzungen zu weiteren Zielgruppen und Lebenswelten
*Abschließend*	
19)	Gibt es noch etwas, was aus Ihrer Sicht relevant ist?
20)	Welche Handlungsempfehlungen möchten Sie zusammenfassend formulieren? (bis zu drei Statements)

Die Auswahl der Teilnehmenden basierte nach einem Schneeballverfahren. Rekrutiert wurden Wissenschaftlerinnen und Wissenschaftler, die im Bereich Ernährung und Ernährungsbildung national und international publizierten sowie Akteurinnen und Akteure entsprechender Praxisprojekte auf Basis der oben genannten Recherche und Handsuche. Anschließend erfolgte eine Abstimmung mit der Steuerungsgruppe. Auf diese Weise wurden 40 Expertinnen und Experten identifiziert, von denen sich 14 zu einem Interview bereit erklärten. Grund für eine Ablehnung waren mangelnde Bereitschaft, Zeitmangel oder eine ausbleibende Antwort der eingeladenen Personen.

Die interviewten Personen übten die folgenden Berufe aus: wissenschaftliche Tätigkeiten an verschiedenen Universitäten/Instituten (TU München, ethnomedizinische Institut Hannover, Uni Mainz) *n* = 3, Regional-/Projektkoordination/Leitung („Da Migra“, „GeMuKi“ und Amt für soziale und psychologische Dienste Offenburg) *n* = 3, Ökotrophologie *n* = 2, Berufsschullehre für Pflegeberufe *n* = 2, Gesundheits-, Familien, Kinderkrankenpflege sowie Tätigkeiten bei den Frühen Hilfen *n* = 2, Still- und Krisenberatung *n* = 1, Ernährungs- und Gesundheitswissenschaft *n* = 1. Teilweise verfügten sie über Zusatzqualifikationen und -tätigkeiten wie Aus-, Fort- und Weiterbildung von Hebammen/Pädagoginnen und Pädagogen/Ökotrophologinnen und Ökotrophologen* n* = 2, Adipositastrainerinnen oder Adipositastrainer *n* = 1, Vorstandsarbeit *n* = 1, Präventionsassistentinnen und Präventionsassistenten *n* = 1, Familienassistentinnen und Familienassistenten* n* = 1.

### Leitfadengestützte Interviews

Durchgeführt wurden die Leitfadeninterviews zwischen Dezember 2020 und April 2021 nach dem SPSS-Prinzip von Helfferich [16]. Neben 1) „Einleitenden Fragen zur Person“ lag der Schwerpunkt auf 2) der „Einschätzung der allgemeinen Situation/Handlungs- und Versorgungsbedarf im Kontext der fokussierten Zielgruppen“, 3) „Fragen zur Ausgestaltung von Programmen/Ideen für Versorgungsziele/-strategien“, 4) „Fragen zur Erreichbarkeit vulnerabler Gruppen“ und 5) „abschließende Fragen“. Inhaltlich wurden die Personen auf Basis 18 offen gestellter Fragen zu einem möglichen Handlungs-/Versorgungsbedarf im Hinblick auf: Kriterien/Ansätze erfolgreicher Interventionen/Maßnahmen zur Ernährungsgesundheit bzw. ernährungsfördernde (kommunale) Strukturbildung sowie Vorschläge für Versorgungsziele, Versorgungsstrategien/-intervention auf Basis des § 20a SGB V befragt (s. Tab. 1).

### Untersuchungsdurchführung und -analyse

Die Teilnehmenden der Interviews wurden über deren Ziele, das weitere Vorgehen und ihre Rechte informiert. Zunächst wurden eine schriftliche Einwilligung zur Teilnahme und eine datenschutzrechtliche Erklärung eingeholt.

Die Interviews wurden digital aufgezeichnet, vollständig transkribiert und die personenbezogenen Angaben anonymisiert. Die Transkripte wurden anschließend nochmals zur Durchsicht und für eventuelle Korrekturen an die Teilnehmenden übersandt. Beiträge konnten auch widerrufen werden.

Anschließend wurden die Transkripte mit MAXQDA 2020 (VERBI – Software. Consult. Sozialforschung. GmbH, Berlin, Deutschland) entsprechend Best-practice-Methoden für qualitative Studien verwaltet und analysiert [27]. Die Auswertung der qualitativen Daten erfolgte anonymisiert zunächst deduktiv durch die Erstellung eines Kategoriensystems aus den vorgegebenen Fragen des Interviewleitfadens [12]. Dabei wurden fünf Oberkategorien gebildet: Einschätzung der allgemeinen Situation/Handlungs- und Versorgungsbedarf, Ausgestaltung von Angeboten, Zugangswege, Netzwerk und Akteurinnen und Akteuren, Handlungsempfehlungen. Die einzelnen Fragen des Leitfadens bildeten die Unterkategorien und wurden den entsprechenden Oberkategorien zugeordnet (Tab. 2). Das entwickelte Kategoriensystem wurde im Anschluss auf die Transkripte angewandt. Die Antworten durften mehreren Kategorien zugeordnet werden. Anschließend wurden die kodierten Daten für die weitere Bearbeitung von MAXQDA in MS Word (Microsoft Corporation® Redmond, WA, USA) heruntergeladen. Im nächsten Schritt erfolgte die Paraphrasierung der Aussagen und deren Generalisierung sowie die Auszählung und tabellarische Zusammenfassung der Antworten anhand des Kategorienschemas.

**Tab. 2:** Kategorienschema der Oberkategorien I, II, III, IV

**Oberkategorie I: Einschätzung der allgemeinen Situation sowie des Handlungs- und Versorgungsbedarfs**			
*1) Allgemeine Gründe für die Relevanz von Präventionsprogrammen*	*2) Einschätzung der aktuellen primärpräventiven Versorgung hinsichtlich der Förderung der Ernährungsgesundheit in den ersten 1000 Tagen*		
**Oberkategorie II: Ausgestaltung von Angeboten**			
*I) Konzeption*	*II) Praktische Umsetzung*	*III) Inhalte*	
II.1) Erfolgsfaktoren	II.1) Allgemein	III.1) Übergeordnete Themen	
II.2) Barrieren	II.2) Gruppen und Familien in belasteten Lebenslagen	III.2) Spezifische Inhalte	
	II.3) Explizit für Kinder	III.3) Entwicklungspsychologische Aspekte	
	II.4) Medien		
**Oberkategorie III: Zugangswege**			
*A) Lebenswelten*	*B) Gruppen und Familien in belasteten Lebenslagen*		
A.1) Wohnumfeld	B.1) Allgemein		
A.2) medizinische- und pflegerische Versorgung	B.2) Medizinische- und pflegerische Versorgung		
A.3) Lernumfeld/Bildungsstätten	B.3) Wohnumfeld		
	B.4) Lernumfeld/Bildungsstätten		
	B.5) Freizeitgestaltung		
**Oberkategorie IV: Netzwerk/Akteurinnen und Akteure**			
*a) Rahmenbedingungen*	*b) Benötigte Partnerinnen und Partner/Netzwerk*	*c) Verbesserung der Nachhaltigkeit*	*d) In den verschiedenen Lebenswelten*
a.1) Bzgl. Netzwerk/Akteurinnen und Akteure	b.1) Allgemein	c.1) Allgemein	d.1) Lernumfeld/Bildungsstätten
a.2) Bzgl. Kommunen	b.2) Medizinische und pflegerische Versorgung		d.2) Medizinische und pflegerische Versorgung
a.3) Bzgl. Politik			

## Ergebnisse

Die Ergebnisse der Recherche der Praxisprojekte auf kommunaler Ebene sind in den Tabellen S1 (*n* = 19 Einschluss) bzw. S2 (*n* = 8 Ausschluss) im Anhang unterteilt nach den jeweiligen Zielgruppen zusammengefasst. Dargestellt wurden die relevanten Kenngrößen der Programme sowie Kernergebnisse. Maßgeblicher Ausschlussgrund war, dass die Zielgruppe nicht die ersten 1000 Tage betraf.

### Leitfadeninterviews

**Abb. 1:**
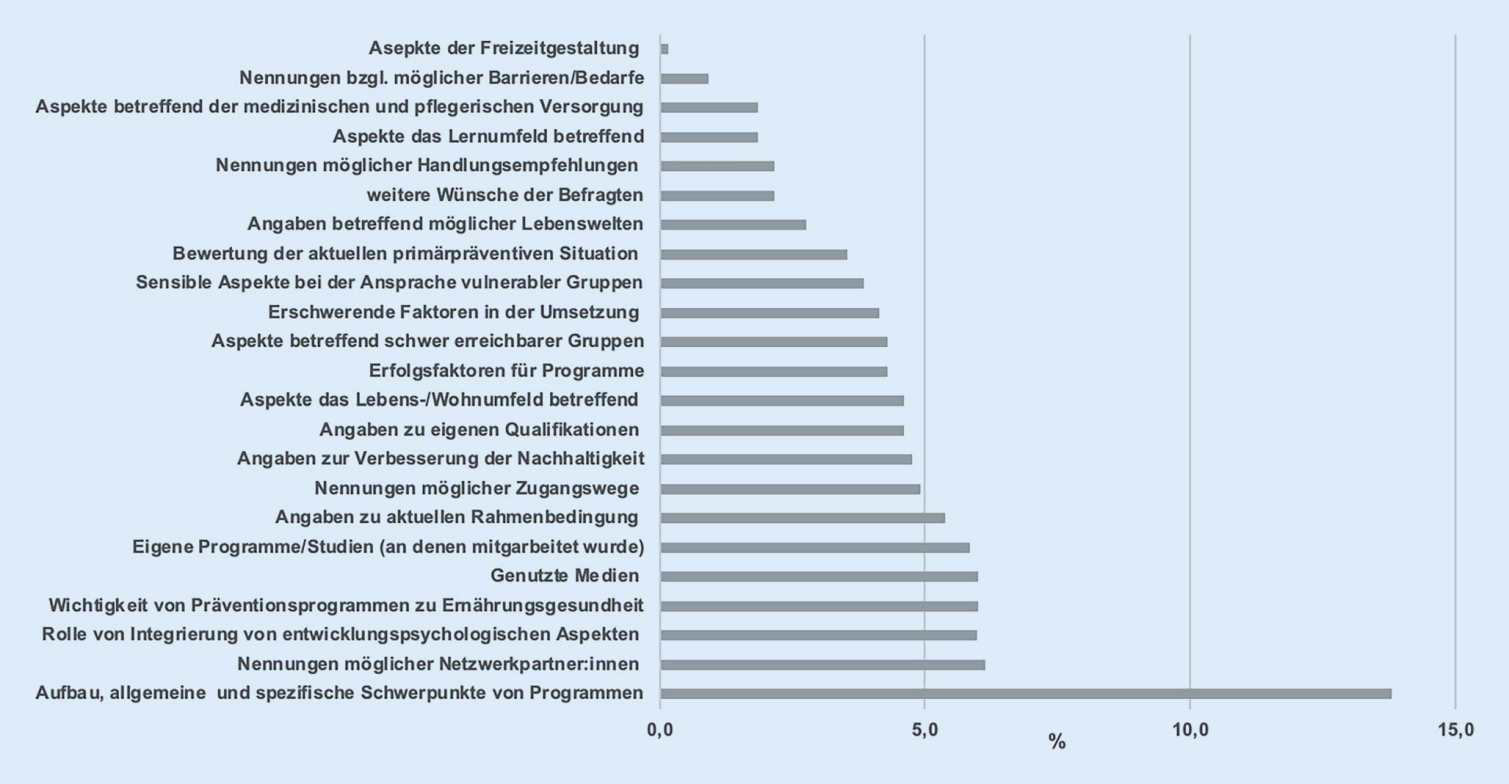
Häufigkeiten der Antworten pro Kode in Prozent (inhaltlich passende Angaben wurden zusammengefasst)

Die Interviewlänge variierte zwischen 30 und 92 min. Die Durchschnittslänge betrug 52,3 ± 18,4 min. Das gesamte Interviewmaterial umfasste 737 min. Daraus ergaben sich 651 Kodierungen. Die Häufigkeiten der genannten Antworten pro Kode sind in Abb. 1 dargestellt. Eine Zusammenfassung der zentralen Ergebnisse der Oberkategorien I, II, III, IV finden sich in Tab. 3. Die Ergebnisse der Oberkategorie V wurden stichpunktartig in Tab. 4 aufgeführt. Die in Klammern angegebenen Zahlen geben an wie oft die Antwort von den Expertinnen und Experten genannt wurden.

**Tab. 3:** Modifizierte Ergebnisse der Oberkategorien I, II, III, IV

**Oberkategorie I: Einschätzung der allgemeinen Situation sowie des Handlungs- und Versorgungsbedarfs**			
*1) Allgemeine Gründe für die Relevanz von Präventionsprogrammen* – zur Prävention von nicht übertragbaren Erkrankungen – um allgemeine gesundheitliche Chancengleichheit zu schaffen – da Fehl- und Überernährung durch die Lebensbedingungen und das -umfeld gefördert wird – da die Primärprävention auch aus ökonomischer Sicht sinnvoll ist	*2) Einschätzung der aktuellen Versorgungslage* *Nicht ausreichend, weil:* – die Aufklärungsaktivitäten weitestgehend erfolglos sind – das Angebot nicht systematisch, flächendeckend und vernetzt erfolgt – Programme wenig vorhanden bzw. sichtbar sind – eine Einbindung in die Lebenswelten fehlt – keine flächendeckende Zusammenarbeit mit den Krankenversicherungen erfolgt – das neue Präventionsgesetz die Durch- und Weiterführung eines Projekts erschwert – die Zielgruppen durch mangelnde bzw. falsche Informationen/Werbung nicht erreicht bzw. beeinflusst werden *Besonders zu unterstützende Zielgruppen von Präventionsprogrammen:* – Gruppen und Familien in belasteten Lebenslagen, z. B. Menschen mit Migrationshintergrund – schwangere Frauen – Kinder		
**Oberkategorie II: Ausgestaltung von Angeboten**			
*I) Konzeption*	*II) Praktische Umsetzung*	*III) Inhaltliche Ausgestaltung*	
II.1) Erfolgsfaktoren *Planung:* – vor Beginn Bedarfsanalyse → was benötigen die Teilnehmenden? – bestehende Angebote mit einbeziehen – vor Beginn Ressourcenanalyse → was können die Durchführenden leisten, was bringen sie mit? – vorhandene Modelle nutzen *Durchführung:* – einfach zugänglich → kostenfrei, geringer bürokratischer Aufwand – inklusiv, divers – Teilhabe ermöglichen, partizipativ – ressourcenorientiert, zielgruppenspezifisch, individuell – frei von Stigmatisierung – Beachtung des sozioökonomischen Status, Berührungsängste vermeiden, niederschwellig – aufsuchend – Einbindung in die Lebenswelt (Setting-Ansatz), wohnortnah – flächendeckend – systematisch, strukturiert – kontinuierliche Begleitung – langfristige Angebote – offensichtlicher Nutzen sollte erkennbar sein – verschiedene Herangehensweisen nutzen – gute Bewerbung – Personalmanagement (genug Personal, angemessene Entlohnung) Evaluation: – wissenschaftliche Evaluation II.1) Barrieren – Eigenanteil – „hochschwellig“ – fehlende Integrierbarkeit in den Alltag – mangelnde Diversität – Eigeninitiative der Betroffenen – Stigmatisierung – Bevormundung in Form von mangelnder Partizipation, Zusammenarbeit mit den Teilnehmenden – kurze Projektlaufzeit – fehlende Struktur/Systematisierung – wenn Triggerpunkte nicht beachtet werden	II.1) Allgemein – Spaß am Thema vermitteln – motivieren zu einer ausgewogenen Ernährung – Kleingruppen – alltagsnah – praktische Inhalte (gemeinsames Kochen, Bewegen) – Kombination Praxis und wissenschaftliche Theorie – Ansprache auf Augenhöhe durch eine angemessene Kommunikation – mehrsprachig zur Überwindung möglicher sprachlicher Barrieren – leicht verständlich, ggf. mit Bildern arbeiten – Austausch zwischen Teilnehmenden ermöglichen und auch um das Schneeballprinzips zur Wissensvermittlung anwenden zu können – Bindung zwischen Teilnehmenden und Übungsleitenden aufbauen – Verstärkerangebote, z. B. Giveaways	III.1) Übergeordnete Themen: – *Ernährungsweise/-verhalten:* natürliche Ernährung fokussieren, selbstständiges Zubereiten (Essen und Trinken), geregelte Mahlzeitstruktur – *Ernährungswissen:* mit besonderem Fokus auf dem Stillen, zur Schaffung der Basis (überwiegende Wissensvermittlung wird als Barriere gesehen) – *Ernährungskompetenz, wie?* – Allerdings wird Gesundheitsförderung als multimodales Konzept gesehen, sodass die Begriffe teilweise als schwer trennbar angesehen wurden, da sie aufeinander aufbauen	
	II.2) Gruppen und Familien in belasteten Lebenslagen – lebensnah – praktisch – kleinschrittig – unterstützen, nicht ändern – keine Bevormundung, keine Belehrung – wertschätzende empathische Kommunikation, passende Ansprache – Sensibilität – Beachtung der Bedürfnisse – keine Überforderung – multikulturell	II.2) Spezifische Inhalte – Spaß an einer ausgewogenen Ernährung fördern – Steigerung des Selbstwerts/der Selbstwirksamkeit der Teilnehmenden – Nutzen der Maßnahme vermitteln – Frauen in der Schwangerschaft hinsichtlich der Vorteile des Stillens beraten *Spezifische Inhalte im Lernumfeld/Bildungsstätte Kita* – Personal bezüglich des Essverhaltens von Kindern und dem Umgang mit Regeln schulen. Aber Überforderung des Personals durch Mehrarbeit vermeiden – gemeinsame Mahlzeiten fördern – praktische Ernährungsbildung etablieren – den Eltern den Druck nehmen alles richtig machen zu müssen	
	II.3) Explizit für Kinder – spielerisch – praktisch – kindgerecht	III.3) Zu Berücksichtigende entwicklungspsychologische Aspekte – Lebensphasenübergänge sollten begleiten/beachtet werden – könnten zur Beziehungs- und Bindungsförderung genutzt werden – stellen eine Form der Frühförderung dar – bieten einen guten Zugang, da: I) alle sich ein gesundes, altersentsprechend entwickeltes Kind wünschen, II) Essen als angeborener Automatismus zu verstehen ist, aber durch die stark einflussnehmende Umgebung überlagert wird, III) sich auf die Modelle des Lernens (Lernen am Model nach Bandura) bezogen werden könnte, IV) Ernährung körperliche Entwicklung beeinflusst	
	II.4) Eingesetzte Medien: – analoge Medien (Flyer, Broschüren, Bücher, Poster etc.) in Verbindung mit persönlicher Ansprache, in leichter Sprache, in Bildsprache, alltagsnah – digitalen Medien (Apps, Tweeds, Videos, Internet, Posts, Fernsehen, Influencer) auch um Kontakt zur Zielgruppe herstellen zu können → wenig Text, kurze und knackige Infos, Bildung und Unterhaltung verknüpfen – persönliche Ansprache → angepasste Sprache, auf Augenhöhe, auf hoher Vertrauensbasis, durch Mediatorinnen und Mediatoren, durch Fachkräfte, unter Nutzung verschiedener Medien		
**Oberkategorie III: Zugangswege**			
*A) Lebenswelten*			
A.1) Wohnumfeld – Eltern-/Familienzentren – religiöse Einrichtungen – Mutter-Kind-Stiftungen *Spezifisch bei Gruppen und Familien in belasteten Lebenslagen:* – Familienzentren – Einkaufszentren – Jugendamt – Schuldnerberatung – in den eigenen Wohnungen			
A.2) medizinische- und pflegerische Versorgung – Ärztinnen und Ärzte → Bewerbung von Angeboten bzw. Ansprache der Themenschwerpunkte bei U-Untersuchungen – Geburts-/Kinderkliniken (über Babylotsen oder Elternschulen anbieten) – Beratungszentren für Frauen mit Kinderwunsch – Präventionsbeauftragte – Einrichtung von übergeordneten Institutionen/Personen, die Diagnostik durchführen und zielgruppenspezifische Programme vermitteln *Spezifisch bei Gruppen und Familien in belasteten Lebenslagen:* – Gesundheitsamt – Krankenversicherungen – Schwangerenberatungsstellen – Geburtsvorbereitungskurse – Familiengesundheitspflegerinnen und Familiengesundheitspfleger, -hebammen, Stillberaterinnen und Stillberater → Besuche zur Geburt – Frühe Hilfen – weitere Angebote des Gesundheitswesens, z. B. Ernährungsberatungsstellen			
A.3) Lernumfeld/Bildungsstätten – Familienbildungsstätten – Spiel- und Krabbelgruppen – Kita, Tagespflegepersonen → Angebote für Eltern *Spezifisch bei Gruppen und Familien in belasteten Lebenslagen:* – Babykurse – Schule – Bildungseinrichtungen für Erwachsene (wie z. B. VHS) – Ausbildungszentren			
A.4) Freizeitgestaltung *Spezifisch bei Gruppen und Familien in belasteten Lebenslagen:* – kulturelle Vereine – Stadtteiltreffs – religiöse Einrichtungen – kulturelle Migrantenorganisationen – Kulturgeschäfte			
**Oberkategorie IV: Netzwerk/Akteurinnen und Akteure**			
*a) Rahmenbedingungen*	*b) Mögliche Partnerinstitutionen/Netzwerk*	*c) Verbesserung der Nachhaltigkeit*	*d) In den verschiedenen Lebenswelten*
a.1) Bzgl. Netzwerk/Akteurinnen und Akteure: – Netzwerk bilden/Vernetzung von Angeboten und Akteurinnen und Akteure → viele Anlaufstellen ermöglichen, Zentralisierung, Familien zusammenführen – Angebote aufeinander abstimmen – Interdisziplinäre Zusammenarbeit mit Fachgesellschaften, Universitäten und der Politik fördern – krankenversicherungsübergreifende Angebote schaffen – feste Strukturen mit festen Ansprechpartnerinnen und Ansprechpartner – der Aufbau einer Kette, der auf alle Entwicklungspunkte/Lebenslagen eines Kindes übergreift, wird als sinnvoll erachtetet, also ähnlich wie der Ansatzpunkt der Präventionsketten	b.1) Allgemein – Politik auf Bundesebene – Kommune – vorhandene Netzwerke/Träger vor Ort – Fachberatungen der Jugendhilfe/Jugendamt – (Sozial-) Pädagoginnen und Pädagogen, ARGE, freie Träger, Stadtteilmanagerinnen und Stadtteilmanager – Integration von Ehrenamt – Stiftungen	c.1) Allgemein – das Angebot der Krankenversicherung an den Interessen der Mitglieder orientieren – Zusammenarbeit von Kommunen und GKV – Etablierung der Angebote durch den Staat – Schaffung von Kontinuität	d.1) Lernumfeld/Bildungsstätten *Kita:* – Einbindung in die Ausbildung von Erzieherinnen und Erzieher – Fort- und Weiterbildung des Personals – Ernährungsbildung in Kitas und Schulen einführen – Optimierung der Verpflegung
a.2) Bzgl. Kommunen: – kommunale Strukturen etablieren/ermöglichen – kommunale gute Planung – Gesundheitsplanung – sinnvolle kommunale Planung → Bedarfsanalyse erforderlich – große runde Tische auf kommunaler Ebene – Gesundheitsberichterstattung	b.2) Medizinische- und pflegerische Versorgung – Krankenversicherungen – Gesundheitsämter – Familienhebammen/-hilfen – Psychologinnen und Psychologen, Psychotherapeuten		d.2) Medizinische- und pflegerische Versorgung – es sollte ein Programm geben, dass standardmäßig wie eine Vorsorgeuntersuchung für alle angeboten wird → Einbindung in das Gesundheitssystem – Programme in Verbindung mit einer Aufwandsentschädigung also z. B. bei Schwangeren kostenlose IGeL-Leistungen, anbieten – Einbindung in die Ausbildungen von Hebammen – Fort- und Weiterbildung
a.3) Bzgl. Politik: – Überarbeitung des Präventionsgesetzes – Finanzierung regeln – Regelfinanzierung – die Lebensmittelindustrie und deren Werbung reglementieren/regulieren, um den Einfluss zu verringern und die Verbreitung von Fake news einzudämmen → wirtschaftliche Aktivitäten hintenanstellen			

*TN* Teilnehmende, *IGeL* individuelle Gesundheitsleistungen, *GKV* Gesetzliche Krankenversicherung, *VHS* Volkshochschule

### Einschätzung der allgemeinen Situation sowie des Handlungs- und Versorgungsbedarfs im Kontext der fokussierten Zielgruppen

Generell bestand bei allen Befragten Konsens bzgl. der Notwendigkeit früher präventiver Maßnahmen. Gerade innerhalb der ersten 1000 Tage könne durch entsprechende Maßnahmen der Grundstein für eine gesunde Entwicklung im Sinne der perinatalen Prägung gelegt werden. Denn der heutige Lebensstil, insbesondere Fehl- und Überernährung sowie Bewegungsmangel, begünstige die Entwicklung von mütterlichem und väterlichem Übergewicht verbunden mit den entsprechenden Folgeerscheinungen auch für das Un- bzw. Neugeborene. Präventive Maßnahmen seien daher auch aus ökonomischer Perspektive zur Einsparung möglicher Folgekosten sinnvoll.

Allerdings sei die Angebotslage unzureichend und nicht flächendeckend.

„Nun, die [Angebotslage] ist natürlich sehr, sehr bescheiden. Es gibt Aufklärungsaktivitäten, die aber, wie wir wissen, weitgehend erfolglos sind. Man braucht sicher kombinierte Ansätze mit Verhältnisprävention, also sprich, wir müssen die Umwelt, wo immer es geht, so verändern, dass dann auch eine gesunde Ernährung leichter gemacht wird.“ (Interview VIII (H), Pos. 40)

So bestünden Versorgungslücken auf politischer bzw. struktureller Ebene, z. B. in Form der starren Vorgaben des Präventionsgesetzes bzgl. des Einsatzes der Mittel sowie Laufzeit und der meist geringen Zusammenarbeit von Anbieterinnen und Anbietern und den Krankenversicherungen. Die nicht vorhandene Transparenz und/oder Vernetzung könne sowohl ein Überangebot, wie auch einen Mangel an speziellen Programmen und Projekten begünstigen. Selten wären außerdem die Maßnahmen in die Lebenswelten der Zielgruppen eingebunden. Dies betreffe besonders die Familien und Gruppen in belasteten Lebenslagen. Darunter wurden von den Expertinnen und Experten neben Familien aus sozial schwachen bzw. wirtschaftlich schwach aufgestellten Regionen (*n* = 13) und/oder Familien mit Migrationshintergrund (*n* = 4) auch „Teenie-“ (*n* = 3) und/oder psychisch erkrankte Eltern (*n* = 1) verstanden. Begründet wurde die erschwerte Erreichbarkeit u. a. mit Sprachbarrieren, mangelndem Verständnis und kulturellen Unterschiede. Diese Barrieren würden zusätzlich durch den ohnehin schon vorherrschenden (Fach)personalmangel und den damit verbundenen Zeit- und Ressourcenmangel verstärkt.

### Ausgestaltung von Angeboten

Generell wurde von den Expertinnen und Experten Gesundheitsförderung und Prävention als multimodales Konzept verstanden, in dem neben allgemeinen Ernährungsempfehlungen und Ernährungswissen auch Ernährungsweisen/-verhalten, -kompetenz und -einstellung vermittelt werden sollte. In der hier fokussierten Altersgruppe sei besonders die Bedeutung des Stillens hervorzuheben. Zudem seien insbesondere bei Eltern Verunsicherungen, wie die Ernährung für ihr Neugeborenes und Kleinkind bestmöglich zusammengesetzt sein sollte, zu beobachten. Grund dafür seien, dass das Informationsangebot an Eltern …

„… stark überlastet [sei] und sozusagen gestört wird durch moderne Medien wie soziale Netzwerke, das Internet, Aktivitäten jeglicher Art, wodurch es noch schwieriger wird sich wirklich fachlich zu informieren.“ (Interview VII [H], Pos. 40)

Im Kontext Ernährungsweise/-verhalten sei für die Kinder/Familien das Erlernen selbständig zubereiteter Mahlzeiten wichtig. Wünschenswert sei es, die Nährstoffzufuhr/-menge im Sinne des intuitiven Essens wieder an das Sättigungs- und Hungergefühl anzupassen. Darüber hinaus habe die Vermittlung von geregelten Mahlzeitenstrukturen einen hohen Stellenwert. Denn gemeinsame Mahlzeiten am Esstisch würden aufgrund der veränderten Alltagsstrukturen vermehrt wegfallen. Nach der „Structured-day“-Hypothese führe eine geregelte Tagesstruktur jedoch zu einem verbesserten Gesundheitsverhalten bei Kindern und Jugendlichen (vgl. [25]).

Auch die Einbindung von entwicklungspsychologischen Aspekten stuften alle Befragten als sinnvoll ein. Dadurch sei es einerseits möglich, die Lebensphasenübergänge zu beachten und u. a. das Essverhalten adäquat anzupassen. Viele Eltern seien außerdem daran interessiert, dass ihr Kind sich altersgemäß und „gesund“ entwickelt, sodass dieser Aspekt einen guten Zugangsweg zur fokussierten Zielgruppe böte.

Losgelöst von der Vermittlung inhaltlicher Schwerpunkte zur Ernährung sei es besonders für Familien und Gruppen in belastetet vulnerablen Lebenslagen wichtig, im Rahmen möglicher Maßnahmen flankierend auch das Selbstwertgefühl und die Selbstwirksamkeit der Eltern zu steigern.

„Das [unausgewogene Ernährung] hat eigentlich gar nichts mit dem Ernährungswissen zu tun, sondern das hat einfach was mit dem Selbstwert der Menschen zu tun. […] Es ist eben auch dieses Gefühl, wie sorge ich für mich, was mache ich und was ist mein Selbstwert und das hängt nicht an der Ernährung, sondern ist eine Grundeinstellung. Das ist leider besonders ausgeprägt bei Menschen vulnerabler Gruppen […], die überall vermittelt bekommen: ‚du bist nichts wert‘, und ‚was du tust, ist auch nichts wert‘.“ (Interview IV (H), Pos. 109)

Als Grundlage für die Ausgestaltung von Maßnahmen könne der Public Health Action Cycle (PHAC) dienen. Konkret bedeutete dies, dass zunächst eine Bestandsaufnahme bestehender Angebote durchgeführt werden solle. Dieses Vorgehen ermögliche die Detektion von spezifischen Bedarfen der Zielgruppe. Darauf aufbauend könnten und sollten partizipativ zielgruppenspezifische (*n* = 4), individuelle (*n* = 4) und ressourcenorientierte Angebote konzipiert werden. Diese sollten insbesondere für Gruppen und Familien in belasteten Lebenslagen niederschwellig (*n* = 6) und einfach zugänglich sein. Beispielhaft wurden eine kostenfreie Teilnahme und ein geringer bürokratischer Aufwand bzw. Zugang gefordert. Vorgeschlagen wurde außerdem, die Angebote in kleinen Gruppen durchzuführen. Kleinere, ggf. homogenere Gruppen ermöglichten, auf die individuellen Bedürfnisse der Teilnehmenden einzugehen. Inhalte aus Theorie und Praxis sollten möglichst alltagsnah und leicht verständlich vermittelt werden. Wo immer möglich, sollte der Praxisbezug hergestellt werden bzw. eine praktische Ausgestaltung (*n* = 6), z. B. in Form von gemeinsamem Kochen und Einkaufen, erfolgen. Dabei sollten sowohl analoge (*n* = 4; z. B. Broschüren, Bücher, Flyer und Plakate) als auch digitale Medien (*n* = 14; z. B. Apps, Videos, Internet, Influencer, Fernsehkampanien und Feeds) in Kombination mit einer persönlichen Ansprache (*n* = 7) genutzt werden. Auf der anderen Seite könne der gezielte Einsatz digitaler Medien unter Berücksichtigung der oben genannten kritischen Punkte aufgrund der weit verbreiteten alltäglichen Nutzung und geringen Schwelle sinnvoll sein, weil Familien in belasteten Lebenslangen gut zu erreichen sein.

„[…] viele sind in den sozialen Medien unterwegs. Da ist es wichtig, dass das [Videos, Informationen, Tweets] alles nicht zu lang ist, also kurze Impuls-Videos, die werden dann nämlich auch von Anfang bis Ende angeschaut. Unterhaltung ist das da das Stichwort: Wir müssen einen Zugang finden, womit wir die Leute unterhalten und bilden.“ (Interview IX (K), Pos. 100).

Zusätzlich wurde der Einsatz von Verstärkerangeboten sowie kleinen „Giveaways“ als sinnvoll eingestuft. Zum Beispiel wurde vorgeschlagen, die Teilnahme an einem primärpräventiven Angebot mit der Kostenübernahme von IGeL-Leistungen bei Gynäkologinnen und Gynäkologen zu „entlohnen“. Dies wurden z. B. in der GeliS-Studie umgesetzt, in der die Teilnahme mit einem zusätzlichen Test zur Diagnose eines Gestationsdiabetes belohnt und dadurch eine erhöhte Teilnahmebereitschaft beobachtet wurde.

Für die erfolgreiche Umsetzung des/eines Konzepts sei es wichtig, zwischen den Teilnehmenden und den Kursleitenden eine Vertrauensbasis aufzubauen. Im Sinne der wertschätzenden Kommunikation wurde eine empathische Ansprache auf Augenhöhe (*n* = 10) frei von Stigmatisierungen und Belehrungen gefordert. Zur Überwindung möglicher Sprachbarrieren sei – wenn möglich – auf Mehrsprachigkeit der Multiplikatorinnen und Multiplikatoren zu achten. Zusätzlich wurden inklusive, diverse und partizipative Ansätze als Erfolg versprechend eingestuft, z. B. in dem die Programmausgestaltung mit den Teilnehmenden gemeinsam (weiter)entwickelt wird. Darüber hinaus wurde eine fortlaufende wissenschaftliche Begleitung, um die Maßnahmen im Sinne des PHAC ggf. zu optimieren oder die Grundlage für eine Verstetigung zu haben, gefordert.

Auf Seiten der Anbieterinnen und Anbietern sei ein Arbeitnehmerinnen- und Arbeitnehmer-freundliches Personalmanagement, inklusive eines ausreichenden Personalschlüssels und einer angemessenen Entlohnung, eine Grundvoraussetzung für eine gelingende Umsetzung.

### Zugangswege

Generell zeigen sich Schwangere und junge Eltern gegenüber gesundheitsförderlichen Botschaften eher offen. Eine Herausforderung bleibe aber der Zugang zu belasteten Familien und Gruppen. Bewährt hätten sich insbesondere aufsuchende, in die Lebenswelt eingebundene (*n* = 6), wohnortsnahe Konzepte (*n* = 6). Diese sollten flächendeckend und systematisch strukturiert sein, z. B. wie in den Konzepten von Präventionsketten („BEAGLE“, „HAG“, „Mo.Ki unter 3 frühes Fördern von Anfang an“). Zusätzlich sei eine gute und sinnvoll eingesetzte Bewerbung von Angeboten unabdingbar. Vorgeschlagen wurde, dass auf Angebote zielgruppengerecht unter Zuhilfenahme verschiedener Medien (und Sprachen) z. B. durch Tweets, Posts aber auch Flyer, Broschüren und v. a. durch die persönliche Ansprache aufmerksam gemacht wird.

Auch eine Anknüpfung an bestehende Gesundheitssysteme stelle einen Erfolg versprechenden Zugang dar. Personal des Gesundheitswesens, insbesondere Ärztinnen und Ärzte, aber auch Hebammen könnten gut als Türöffner fungieren. Gruppen und Familien in belasteten Lebenslagen seien zudem über Schwangerenberatungsstellen, Geburtsvorbereitungskurse, Gesundheitsämter, Krankenversicherungen, die Frühen Hilfen und über Angebote in Geburtsklinken (z. B. Babylotsen oder Elternschulen) zu erreichen. Weitere Möglichkeiten zur Förderung der Ernährungsgesundheit böten Besuche zur Geburt eines Kindes durch Familiengesundheitspflegerinnen und Familiengesundheitspfleger, -hebammen und/oder Stillberaterinnen und Stillberater (s. Tab. 3).

„Für mich ist zum Beispiel die Schuldenberatungsstelle sinnvoll, wenn man die bei den Familienzentren andockt, wenn man die Beratungsstellen dort vor Ort mit unterbringt, dann ist das im Sozialraum fußläufig wunderbar erreichbar und dann ist das auch so ein Treffpunkt, wo es immer noch gute Einbindungsmöglichkeiten gibt.“ (Interview VI (T-B), Pos. 102).

### Netzwerk/Akteurinnen und Akteure

**Abb. 2:**
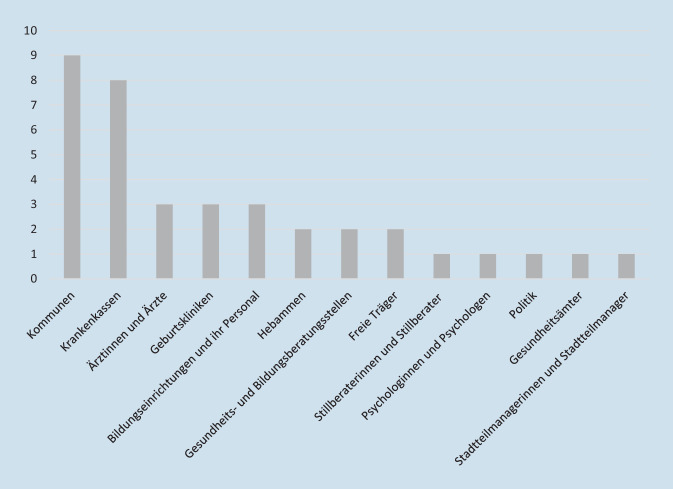
Mögliche Partnerinnen und Partner in (kommunalen) Netzwerken

Als Grundlage für eine flächendeckende Versorgung wurde von allen Befragten das Vorhandensein eines entsprechenden Netzwerks unter Beteiligung relevanter Akteurinnen und Akteure wenn möglich unter Beteiligung kommunaler Stakeholder bzw. Politik und Wissenschaft – benannt (s. Nennungen in Abb. 2). Aufgabe eines solchen Netzwerks sei es, Rahmenbedingungen für das Gelingen primärpräventiver Angebote zu schaffen. So sei es möglich, entsprechende Maßnahmen transparent zu machen, aufeinander abzustimmen und feste Strukturen zu etablieren. In diesem Zusammenhang wurde der Ansatz von Präventionsketten als vielversprechend eingestuft. Bislang gebe es aber in Deutschland nur wenige Präventionsketten in die das Thema Ernährungsgesundheit im Kleinstkindalter, Schwangerschaft eingebettet sei, z. B. „Café Kinderwagen“, „Begrüßungsbesuche bei Familien mit Neugeborenen“, „BEAGLE“, „HAG“, „Mo.Ki unter 3 frühes Fördern von Anfang an“. Aber auch losgelöst davon könnte die Kommune über die kommunale Gesundheitsplanung und Gesundheitsberichterstattung wesentlich zur Ausgestaltung solcher Netzwerke beitragen, z. B. durch die Etablierung „runder Tische“. Die Voraussetzungen aber würden v. a. von politischen Entscheidungen abhängen. (Bessere) Politische Vorgaben zur Regelfinanzierung von Angeboten sowie der flexible Einsatz von Fördermitteln wurden von den Befragten als essenziell für das Gelingen präventiver Maßnahmen eingestuft. Dazu zähle auch die Regulierung und Reglementierung möglicher Einflussnahme durch die Ernährungsindustrie, z. B. die Bewerbung von Kinderlebensmitteln oder mögliche Fake news.

Auch das 2015 verabschiedete Präventionsgesetz (PrävG) bremse eine Implementierung guter Maßnahmen durch sehr starre Vorgaben, was den Einsatz bewilligter Fördermittel anginge, eher aus. Zusätzlich wurde kritisiert, dass überwiegend zeitlich begrenzte Programme existieren. Grund dafür scheinen auch verschiedene (gesetzliche) Vorgaben zu sein. Zum Beispiel wird die Projektlaufzeit von Modellvorhaben im § 20g des PrävG in der Regel auf 5 Jahre begrenzt. Im Leitfaden Prävention des GKV Spitzenverbands ist festgelegt, dass Kurse zur individuellen Verhaltensprävention alle 3 Jahre rezertifiziert werden müssen. Die aufgezeigten zeitlichen Rahmenbedingungen seien oft nicht ausreichend, um nachhaltige Effekte zu erzielen. Schlussfolgernd wurde sich gewünscht, eine flexible zeitliche Ausgestaltung von primärpräventiven Maßnahmen im Präventionsgesetz zu verankern.

Zur Verstetigung von Erfolg versprechenden Angeboten wünschten sich die Expertinnen und Experten eine Kontinuität in der Angebotsstruktur, z. B. durch die intensivere Einbindung von Krankenversicherungen. Sie forderten außerdem, das Angebot der Krankenversicherungen (mehr) an den Interessen ihrer Mitglieder zu orientieren, sodass die Eltern auch hier mehr in ihrem Alltag abgeholt werden könnten. Denn so sei es möglich, dass auch belastete Familien und Gruppen auf die Angebote aufmerksam werden und von diesen profitieren könnten. Außerdem äußerte ein Experte folgenden Wunsch zur Verbesserung der Verstetigung:

„Es müsste wirklich auf gesellschaftlicher Ebene eine konsistente Strategie in allen Lebensphasen verfolgt werden, um eine gesunde Ernährung, eine gesunde Lebensführung zu fördern. Und da haben wir das Problem, dass dies nirgendwo wirklich etabliert ist und hier auch der Staat sich zurückhält und nicht adäquat einbringt.“ (Interview VII (H), Pos. 56)

Außerdem könnte die Integration gesundheitsförderlicher Inhalte in die Ausbildung von Personal im Gesundheitswesen, aber auch Hebammen, Erzieherinnen und Erzieher etc. verankert werden (*n* = 3). Alternativ sollten entsprechende Fort- und Weiterbildungen angeboten werden. Solche Schritte könnten erheblich zur Flächendeckung und Verbreitung präventiver Botschaften beitragen.

**Tab. 4:** Handlungsempfehlungen orientiert an den Angaben der Expertinnen und Experten

Mögliche Handlungsempfehlungen
*Angebote sollten* – für alle Menschen zugänglich sein – aufsuchend, regelfinanziert und kostenfrei sein – sich an den Bedürfnissen der Zielgruppen orientieren – Inhalte sachlich, informativ, in einfacher und verständlicher Sprache und praktisch und unter Nutzung von digitalen Medien vermitteln – evidenzbasiert durchgeführt werden (sofern möglich) – flächendeckend und wohnortnah bzw. in den Lebenswelten der Zielgruppen angeboten werden
*Allgemeine Empfehlungen zu Rahmenbedingungen* – Wirtschaftliche Strukturen sollten mit einbezogen werden – Es sollte eine flexible zeitliche Ausgestaltung von primärpräventiven Maßnahmen etabliert werden, um eine langsame Projektentwicklung mit dem Fokus der Vernetzung verwirklichen zu können – Es sollte sich bewusst gemacht werden, dass die Umsetzung und Erreichbarkeit der Zielgruppen in der Verantwortung der Akteurinnen bzw. Akteure in der Kommune liegt – Es sollten kommunale Präventionsketten genutzt bzw. geschaffen werden – Es sollten zielgruppenspezifische Informationskanäle genutzt werden – Fachwissen sollte durch ausgebildetes/weitergebildetes Fachpersonal vermittelt werden – Die Teilnehmenden sollten befähigt werden, selbstständig fundierte Entscheidungen treffen zu können – Das Personal des Gesundheitswesens sollte die Angebote bewerben bzw. integriert werden

## Diskussion

Schwerpunkt dieser qualitativen Studie lag auf der Versorgungssituation von (kommunalen) Maßnahmen, die sich mit der Förderung der Ernährungsgesundheit in den ersten 1000 Tagen befassen. Auf Basis von Expertinnen und Experten bzw. Experteninterviews wurden deutliche Versorgungslücken, v. a. hinsichtlich der Erreichbarkeit sog. Gruppen und Familien in belasteten Lebenslagen, beschrieben. So fehle für eine erfolgreiche Versorgung die entsprechende Infrastruktur, insbesondere personelle und finanziellen Ressourcen in der Regelversorgung. Kritisiert wurden darüber hinaus die mangelnde Vernetzung und Unterstützung von politischen, kommunalen und anderen relevanten Stakeholdern oder Kostenträgern wie beispielsweise Krankenversicherungen. Auch die Möglichkeiten für Maßnahmenentwicklung im Rahmen des Präventionsgesetzes seien optimierbar, da dies keinen Raum für eine flexiblere Ausgestaltung, u. a. mit Projektgeldern, möglich mache und auch Anschlussfinanzierungen zur Implementierung positiv bewerteter Modelle nur selten gegeben seien. Vorhandene politische Strukturen hingegen (beispielsweise Präventionsketten) schließen diese Zielgruppen und Thematiken eher selten ein. Dabei könnten in den ersten 1000 Tage entscheidende Weichen im Sinne der Gesundheitsförderung und Prävention nicht übertragbarer Erkrankungen u. a. einer gesunden Gewichtsentwicklung gestellt werden [23]. Insbesondere die Lebensmittelwahl im Kleinstkindalter [18, 26] und der BMI der Eltern vor und in der Schwangerschaft gelten als zentrale „early life factors“ bzgl. der metabolischen Gesundheit [8, 11]. Trotz dieser Erkenntnisse zeigen verschiedene Studien, dass die Ernährungssituation der Kinder suboptimal [4, 17] und die Übergewichtsprävalenz bei Eltern mit 28,1 % (Stand Mikrozensus 2017) nach wie vor hoch ist [10, 19, 30]. Die Auswertung des Instituts für Qualitätssicherung und Transparenz im Gesundheitswesen (IQTIG) hat sogar gezeigt, dass 2020 40 % der schwangeren Frauen bei der Erstuntersuchung übergewichtig waren [19]. Bezogen auf die Ernährungsgesundheit werden laut der KiGGS-Welle 2 nur 72 % der Säuglinge voll gestillt. Außerdem nahm das ausschließliche Stillen ab dem 3. Lebensmonat deutlich ab und die empfohlene Dauer von insgesamt 4 Monaten wurde nur von 46 % erreicht [4]. Die Ergebnisse der GRETA-Studie zeigten auch Defizite im Ernährungsverhalten von Kleinstkindern; nur in 60–70 % wurde die empfohlenen Verzehrmengen von pflanzlichen Lebensmitteln eingehalten [17]. Der Fokus sollte daher verstärkt auf diese frühe Phase gelegt werden; leider zeigt die Analyse der Praxisprojekte den größeren Schwerpunkt im Setting Kindergarten. Dabei sind insbesondere schwangere Frauen und junge Eltern offen für gesundheitsfördernde Botschaften. In einer Auswertung des „BabyCare“-Programms in Berlin gaben 62,2 % der 3514 teilnehmenden Schwangeren an, ihre Ernährung umgestellt zu haben und 58,7 % gesundheitsbewusster geworden zu sein [21, 24]. Auch die Etablierung von Erzählcafés für Schwangere hat laut der Initiatorinnen großen Zuspruch erfahren und einen Austausch von werdenden Eltern und die Stärkung deren Eigenkompetenz ermöglicht. Zudem konnte bundesweit auf Missstände in der Geburtshilfe aufmerksam gemacht sowie die Vernetzung Akteurinnen und von Akteuren geschaffen werden [34].

Sicherlich sind diese einzelnen Erfolge begrüßenswert. Um aber eine echte Trendwende zu erreichen, sind politische Änderungen vonnöten. Bereits 2019 wurden in einer Stellungnahme der Bundesregierung zum nationalen Präventionsbericht einige Aspekte des Präventionsgesetztes kritisiert. Dazu zählte u. a. die optimierbare Intensivierung der kommunalen Gesundheitsförderung und Vernetzung mit verschiedenen/anderen Institutionen sowie eine erforderliche Verbesserung der Leistungsangebote für Eltern von Kleinkindern und Schwangere [7]. Notwendig ist auch eine Vereinheitlichung der heterogenen Gesundheitsförderungsstruktur in Deutschland, die sich aktuell durch eine Fragmentierung der Akteurinnen und Akteure und Zuständigkeiten, Vielfalt der Interessen und unzureichenden Koordinierung der Handlungsebenen und Akteurinnen und Akteure auszeichnet [7, 20].

Ein zentraler Punkt war aus Sicht der Befragten auch die Einflussnahme der Ernährungsindustrie bzw. diese adäquat und konsequent zu regulieren und zu reglementieren. International finden sich bereits entsprechende Beispiele. Zum Beispiel wurde 2020 in zwei Bundesstaaten in Mexiko ein Gesetz erlassen, das den Verkauf von zuckerhaltigen Getränken und Fast-Food an Personen unter 18 Jahren verbietet um der hohen Prävalenz von Übergewicht und Adipositas entgegenwirken zu können [28].

Kombinierte Ansätze der Verhältnisprävention und der Verhaltensprävention haben nachweislich eine deutlich höhere Reichweite als (einzelne) verhaltenspräventive Angebote [9]. Diese sollten auch nicht singulär auf ein Themenfeld wie beispielsweise Ernährung fokussieren, sondern alle Säulen der Prävention wie Bewegung/Bewegungsräume, Reduktion vermeidbarer Sitzzeiten, Stressregulierung und Schlaf in den Blick nehmen [1]. Neben der entsprechenden Ausgestaltung der Lebensräume, der Verzahnung entsprechender Angebote in Präventionsketten gilt es insbesondere, die Gesundheitskompetenz zu steigern. Dieser Aspekt betrifft v. a. die sog. Gruppen und Familien in belasteten Lebenslagen, die nicht selten ungünstigere Ernährungsweisen, weniger Bewegung, mehr Mediennutzung, häufiger Übergewicht und Adipositas bzw. nicht übertragbare Erkrankungen aufweisen [31]. Allerdings werden sie durch die herkömmlichen Maßnahmen deutlich schlechter erreicht [32, 33]. Primärpräventive Angebote sollten daher niederschwellig, zielgruppenspezifische, individuell und partizipativ konzipiert werden [36]. Zudem versprechen kommunal basierte Ansätze, die wohnortsnahe, lebensweltorientierte und aufsuchende Angebote umfassen, den größten Erfolg und werden als Goldstandard gesehen [3]. Innovative Herangehensweisen wie Lotsendiensten in Geburtskliniken oder Müttercafés in Familienzentren oder eine engere Anknüpfung an Gesundheitssysteme (z. B. Gesundheitskioske; mod. nach [5]) könnten weitere Lösungsmöglichkeiten bieten. Personal des Gesundheitswesens, u. a. Ärztinnen und Ärzte und Hebammen, können als erfolgreiche Türöffner fungieren, auf präventive Maßnahmen aufmerksam machen oder dorthin überleiten.

Zusätzlich sollte der Bekanntheitsgrad wissenschaftlich fundierter Handlungsempfehlungen und evidenzbasierter Herangehensweisen gesteigert werden und wenn möglich sogar in alle Ausbildungsgänge beteiligter Berufsgruppen wie Erzieherinnen und Erzieher, Hebammen, Gesundheits- und Kinderkrankenpflegerinnen und Gesundheits- und Kinderkrankenpfleger integriert werden. Dadurch sei es auch auf Basis unserer Interviews möglich, einheitliches Wissen zu vermitteln und der vorherrschenden Verunsicherung der Eltern bezüglich einer gesunden Ernährung entgegenzuwirken.

## Stärken und Limitationen

Eine Stärke ist die umfassende Herangehensweise der Analyse. So erfolgte zunächst eine internetbasierte Recherche bzgl. entsprechender wissenschaftlicher, aber auch (kommunal verankerter) Praxisprojekte (s. Anhänge) zur Konzeption der Interviews. Die sich daraus ergebenden Lücken sollten dann mit den Expertinnen-/Experteninterviews geschlossen werden. Viele der Befragten waren schon lange im Bereich der Gesundheitsförderung tätig. Von Vorteil waren daher entsprechende Kenntnisse und Erfahrungswerte; es zeichneten sich aber auch Frustrationen für die geringen Änderungsmöglichkeiten ab. Allerdings waren auch diese wie Interviews i. Allg. geprägt von ihrem subjektiven Charakter. Bedauerlicherweise erklärten sich auch lediglich 14 der 40 eingeladenen Expertinnen und Experten zur Teilnahme bereit. Wir können nur spekulieren, aber möglicherweise hätte eine höhere Anzahl an Befragten aus allen verantwortlichen Sparten zu (noch) deutlicheren Aussagen geführt. Die beruflichen Hintergründe der Befragten waren sehr heterogen, kein einziger kam aus dem politischen Kontext. Abschließend erschwerten die Heterogenität der Antwort und eine nicht immer gegebene Trennschäre der Leitfragen die Kategorisierung und klare Zuordnung zu möglichen Handlungsempfehlungen.

## Schlussfolgerung und Ausblick

Zusammenfassend bestätigen unsere Ergebnisse aus Sicht von Expertinnen und Experten, dass die Förderung der Ernährungsgesundheit in den ersten 1000 Tagen sinnvoll und wichtig ist. Erfreulich ist, dass sich mit dem Koalitionsvertrag zur 20. Wahlperiode bzgl. an Kinder gerichtete Werbung für Lebensmittel etwas zu bewegen scheint. Dieser sieht vor, die Werbung von fettreichen, besonders zucker- und salzhaltigen Lebensmitteln in Sendungsformaten für Kinder unter 14 Jahren zu verbieten [2]. Allerdings besteht trotzdem ein deutlicher Optimierungsbedarf hinsichtlich der Versorgungsstrukturen und der konkreten Umsetzung von primärpräventiven Angeboten.

Bereits vor der COVID-19-Pandemie („coronavirus disease 2019“) waren deutliche soziale Unterschiede zu erkennen; SARS-CoV-2 („severe acute respiratory syndrome coronavirus 2“) hat die gesundheitliche und soziale Schere noch weiter auseinandergetrieben [7, 37]. Umso wichtiger ist daher, die entsprechenden Rahmenbedingungen zu schaffen, aber auch vorhandene Ansätze wie in Präventionsketten oder auch die aktuell neu entstehenden Gesundheitskioske zu nutzen bzw. zu vernetzen (mod. nach [5]).

Zudem sollte die Vernetzung von politischen und strukturellen Stakeholdern und Akteurinnen und Akteure forciert werden, um so eine flächendeckende und nachhaltige Angebotsstruktur insbesondere für diese frühe Phase bieten zu können. Auch innovative Ansätze mit dem Einsatz digitaler Medien sollten forciert werden (mod. nach [6]). Diese Herangehensweisen sollten adäquat wissenschaftlich begleitet werden und sich an vorhandenen Qualitätskriterien (z. B. gesundheitliche Chancengleichheit) und/oder dem PHAC orientieren [13]. Nur so können Erfolg versprechende, aber auch hinderliche Faktoren detektiert und eingesetzt bzw. vermieden werden.

## Fazit für die Praxis

- Es sollte ein Verständnis für die Bedeutung der frühkindlichen Ernährungsgesundheit geschaffen werden.
- Im Zentrum möglicher Maßnahmen sollte die Förderung der Ernährungskompetenz bei werdenden Eltern bzw. jungen Familien stehen.
- Maßnahmen sollten evidenzbasiert, niederschwellig und wohnortnah, ggf. aufsuchend für Schwangere bzw. Familien in belasteten Lebenslagen sein.
- Die Ausgestaltung von Interventionen erfordert eine partizipative Herangehensweise.
- Bewährt haben sich praxisnahe und individualisierte Programme anstelle von beispielsweise Hochglanzbroschüren.
- Zusätzlich ist der zielgruppengerechte Einsatz digitaler Medien empfehlenswert.
- Jegliche Maßnahmen sollten von qualitätssichernden Schritten im Sinne des Public Health Action Cycles begleitet werden.
- Zur Optimierung der aktuell eher lückenhaften Versorgungslage sollten politische Schlüsselpersonen eingebunden und bewährte Programme in kommunale Strukturen, z. B. Präventionsketten, integriert werden.

## Supplementary Information

Tabelle S1 Übersicht über die ausgewählten (kommunal verankerten) Projekte

Tabelle S2 Ausgeschlossene (kommunal verankerte) Projekte
