# Supplementary material for: Community-based care structures to promote nutritional health in the first 1000 days—Analysis of the status quo
Source: Pravent Gesundh. 2022 Nov 28:1–15. [Article in German] Online ahead of print. doi: 10.1007/s11553-022-00998-2 (PMC9707151; doi:10.1007/s11553-022-00998-2)
Supplement: Supplementary file 1 — Tabelle S1 Übersicht über die ausgewählten (kommunal verankerten) Projekte [file 11553_2022_998_MOESM1_ESM.pdf]

Tabelle S1 Übersicht über die ausgewählten (kommunal verankerten) Projekte

Titel	Laufzeit	Träger/Institution	Für Zielgruppe relevante Maßnahme	Ergebnisse	Sonstiges	Quelle
Kommunal-verankerte Projekte mit der Zielgruppe: Schwangere						
Lenzgesund - Vernetzte frühe Hilfen rund um Schwangerschaft, Geburt und erste Lebensjahre	2005-2012	Kooperations-verbund Gesundheitliche Chancengleichheit	Veranstaltungsreihe „Gesundheitsgespräche für Frauen von Frauen“ Familienhebamme, Initiierung eines Gymnastikangebots für Frauen, andere Aktivitäten, wie z.B. Gesundheitsquiz bei Quartiersfesten oder Durchführung eines Impfprojekts im Rahmen der „Nationalen Impfwoche“	- Babys und ihre Mütter/Eltern werden heute besser versorgt und wahrgenommen<br>- Verbesserte Gesundheit und Gesundheitschancen bei Kindern laut Schuleingangsuntersuchungen (geringes Geburtsgewicht seltener, Anteil übergewichtiger Kinder zurückgegangen, deutlich mehr naturgesunde Zähne bei Erstklässlern, starker Anstieg des ganztägigen Kitabesuchs)<br>- Bewohner/-innen sind deutlich bewegungsaktiver geworden<br>- Mehr Einrichtungen haben das Thema Gesundheit in ihr Angebot integriert<br>- Angebote und Maßnahmen wurden in bestehende Strukturen integriert → größere Akzeptanz und Erreichbarkeit der Zielgruppe<br>- je mehr sich das Angebot am praktischen Bedarf der Bewohnerinnen orientierte, desto häufiger wurde Angebot genutzt<br>- Kombination von Infoveranstaltungen mit praktischen und ortsnahen Folgeangeboten versprachen größten Erfolg<br>- „Gesundheitsgespräche für Frauen von Frauen“: Integration der Veranstaltung im Mittwochscafé, einem von deutschen Frauen besuchten Treff → sozial benachteiligte deutsche Frauen wurden besser erreicht	- Gewann 2014 den Qualitätspreis der Akademie für öffentliches Gesundheitswesen	https://www.gesundheitliche-chancengleichheit.de/good-practice/praeventionsprogramm-lenzgesund#:~:text=Das%20Pr%C3%A4ventionsprogramm%20%E2%80%9ELenzgesund%20%E2%80%93%20Vernetzte%20fr%C3%BChe,von%20medizinischen%20und%20sozialen%20Dienstleistungen.
Kommunal-verankerte Projekte mit der Zielgruppe: Familie						
Bille in Bewegung – fit, pfiffig und kooperativ	2006-2009, Oktober 2009-August 2011	Arbeit und Leben Hamburg e. V.	a) „Fachgespräche und -konferenzen“<br>- geplant 3, durchgeführt 3<br>- inhaltlicher Austausch über relevante Aspekte von Bewegung und Ernährung<br>- Gedanken- und Erfahrungsaustausch<br>b) „Gruppenberatung zur Ernährung“	Förderlich:<br>- bereits bestehende Strukturen nutzen und neue Projekte auf diesen Strukturen aufbauen<br>- Koordinationsbüro als Knotenpunkt und Ansprechpartner für alle Projekte kümmerte sich um Koordinierung und		https://www.in-form.de/netzwerk/projekte/bille-in-bewegung-fit-pfiffig-und-kooperativ/

			- Vermittlung zielgruppengerechter und niedrigschwelliger Informationen an junge bzw. werdende Eltern zum Thema gesunde Ernährung für sich und ihr Kind<br>- geplant 4 Kurse, realisiert 3 Kurs	damit zusammenhängende Aufgaben einschließlich der fortlaufenden Dokumentation<br>- Nachfrage größer als Angebot von Maßnahmen; nicht alle Anfragen konnten erfüllt werden<br>- Projektstart fiel zusammen mit Senatsprogrammen der sozialen Stadtteilentwicklung Herstellung wertvoller Synergien und Integration der Projekte in die Kommunalentwicklung<br>- parteiübergreifender politischer Konsens in den Fragen Prävention und Kinderschutz breite politische Unterstützung des Vorhabens durch kommunalpolitische Gremien<br>- aktive Einbindung des Personals von Grundschulen, Kitas sowie kommunaler Familien-, Kinder-, und Jugendeinrichtungen gute Voraussetzung, für angestrebte eigenständige Weiterführung der Aktivitäten nach Projektende<br>- enge Zusammenarbeit mit bedeutenden Schlüsselakteur/-innen auf dem Gebiet gesundheitliche Prävention<br>- Networking der Programmverantwortlichen gute Zugangswege in<br><br>Hemmend:<br>- Ressourcenknappheit bei beteiligten Partnerorganisationen (finanziell und Zeit)<br>- politische Prozesse (Wahlen, Volksentscheid) und Entscheidungswege verzögerten<br>die Arbeit<br>- Unterschätzung des zeitlichen und organisatorischen Aufwands bei Kooperationspartner/-innen, von denen einige wegen zu knapper Budgets absprangen<br>a) „Fachgespräche und -konferenzen“<br>- eklatantes Wissensdefizit bei jungen Müttern (Eltern) hinsichtlich des		

				Umgangs mit ihren Kindern (Anleitungen zum Spiel, Schmusen, Vorlesen usw. wird benötigt) Idee: Elternführerschein<br>- Hoher Bedarf an Familienhebammen und Mütterberatung, für den die vorhandenen Ressourcen nicht ausreichen Ressourcen und Stundenzahl der Kita-Ärztin erhöhen Idee: Konzept wie in den Niederlanden, zwei Schwestern pro Stadtteil, die alle werdenden Eltern besuchen<br>Strukturelles Defizit bei Fach- bzw. Kinderärzt/-innen, Zulassung von Kinderärzt/-innen müsste durch kassenärztliche Vereinigung stärker geregelt bzw. organisiert sein<br>- es fehlen Selbsthilfegruppen<br>- Interesse an Thema Gesundheitsförderung wird häufig erst bei Bedarf geweckt; Besser: Einbeziehung des Themas in bestehende Kursangebote (wohnortnah, vertraute Umgebung), Kinderbetreuung ist unbedingt notwendig<br>- Sportangebote für Migrantinnen werden zunehmend angefragt und sollten verstärkt angeboten werden<br>- Identifizierung und Diskussion folgender Themen: Ernährung/Hunger, Psychosoziales Wohlbefinden und psychische Gesundheit, Bewegungsmangel<br>- Bereich Bewegung und Ernährung: kontinuierliche, fachlich qualifizierte Angebote der Bewegungsförderung und kostenfreie, gesunde Verpflegung für alle Heranwachsenden in Kindertagesbetreuung (und Schulen), ohne dass dies zu Kürzungen an anderen Stellen führt<br>b) „Gruppenberatung zur Ernährung“:<br>- Offenheit der Programmpartner (hier Eltern-Kind-Zentren) für das Projekt und große Flexibilität der Referentin führten auch bei heterogener Gruppe zu einem Erfolg der Maßnahme		

				- unterschiedlicher Erfahrungs- und Kenntnisstand der Teilnehmer/-innen, sowie Sprachenvielfalt innerhalb der Gruppe hätte problematisch werden können wurde sehr gut von Referentin abgefangen<br>- Themen Bewegung und Ernährung konnte erfolgreich in zahlreiche Kindertagesstätten (und Schulen) getragen werden<br>- angelegte Kindergärten bestehen weiter<br>- Kochstationen und Kochfeste wurden wiederholt und in andere Stadtteile getragen		
Das schmeckt gut!	2005-2006	AWO IntegrationsCenter Ost Kiel	- Interkulturelles Ernährungsprojekt, das sowohl Angebote für Eltern als auch die Kinder beinhaltet<br>- Beteiligt waren zweisprachige Fachkräfte, wie Ökotropholog/-innen, Kinderärzt/-innen und Erzieher/-innen<br>- Vorträge und Infoabende für Eltern zu den Themen Süßigkeiten, Kinder und Übergewicht, Frühstück<br>- Anbieten von Eltern-Kind-Nachmittagen, wo gemeinsam Speisen zubereitet werden<br>- vielfältige Bearbeitung und Integrierung des Themas Ernährung in den Kita-Alltag<br>- Maßnahme wurde in Kinderhaus der AWO Kiel durchgeführt<br>a) „Gib Süßes oder es gibt Saures!“ Vortrag und Erfahrungsaustausch für Eltern<br>b) „Durch dick und dünn – Übergewicht bei Kindern“ Informationsabend für Eltern zum Thema Übergewicht bei Kindern<br>c) Eltern-Kind-Nachmittag: Erfahrungsaustausch bei einem Buffet mit gesunden interkulturellen Gerichten	- Ansprache verfolgte über persönliche Gespräche, Aushänge und schriftliche Einladungen in der jeweiligen Muttersprache AWO-Mitarbeiterin rief kurz vor Programmbeginn noch einmal alle Eltern an, um die Teilnahme am Elternabend zu sichern und so niederschwellig wie möglich zu halten<br>- Zweisprachigkeit der Partner/-innen wurde positiv aufgenommen, bei Bedarf konnte in den Elternveranstaltungen übersetzt werden (Senkung der sprachlichen und kulturellen Zugangsschwelle) adäquate Verständigung war möglich, Individuelle Bedürfnisse konnten berücksichtigt werden<br>- Keine Programmkosten, Wartezeiten oder Anmeldeformalitäten<br>- Mitgebrachte Getränke von Eltern, die zuckerhaltig sind, werden zurückgegeben. Stattdessen werden Mineralwasser, Tee, Milch und gelegentlich Apfelschorle für alle Kinder angeboten<br>- Fortbildung der Projektdurchführenden Erzieher/-innen im Bereich Ernährung<br>- wöchentlicher Müslitag<br>- ausgewogeneres Mittagessen mit einem höheren Frischkostanteil		https://www.gesundheitliche-chancengleichheit.de/good-practice/das-schmeckt-gut/
Dortmunder Kinder. Besser essen. Mehr bewegen. (DoKi), „Sağlıklı	21.09.2006 – 30.11.2009	Gesundheitsamt der Stadt Dortmund, gefördert durch das Bundesministerium für	- Aufbau eines Netzwerks, um Angebote zur Adipositasprävention in Schulen,	- Kurse und Beratungen wurden weniger angenommen als geplant	Übergewicht:<br>- Ausweitung der Erziehungsberatung	https://www.in-form.de/netzwerk/projekte/dortmund

büyümek“, „Wyrosnąć zdrowo“, „gesund groß werden“ – das klappt in allen Sprachen		Ernährung, Landwirtschaft und Verbraucherschutz	Kindertageseinrichtungen und im Alltag von Familien zu verankern<br>- Kinder früh in Sportvereine einbinden und die Eltern mit muttersprachlichem Angebot in ihrer Lebenswelt erreichen Zugang zu schwer erreichbaren Familien schaffen:<br>a) Stadtbezirksbezogene Beratung zur Prävention von Übergewicht in den zwölf Familienbüros der Stadt Dortmund<br>- Sammlung aller stadtberzirksbezogenen Angebote zur Übergewichtsprävention individuelles und passgenaues Angebot für Familie/Einrichtung kann so identifiziert werden<br>- Familienbüros stellen Kontakt zu Ansprechpartnern der Angebote her<br>- Maßnahme „Obst und Co. Macht Kind froh” = Infoveranstaltung für Eltern zum Thema gesunde Ernährung<br>- Maßnahme „Bewegungsforscher“ für Eltern und Kinder selbstbestimmte Erkundung einer kreativen Bewegungslandschaft, um vorhandene Fähigkeiten auszubauen und soziale Kompetenz zu stärken<br>- Maßnahme „Essen wie die „Großen““ Infoveranstaltung für Eltern von Säuglingen ab ca. dem 1. Monat zur Vermittlung von Inhalten zu den Themen Stillen und Beikost<br>- Maßnahme „Von der Milch zum Brei“ Ernährungsberatung für das erste Lebensjahr<br>b) Muttersprachliche Elternbegleiter/-innen in der Prävention von Übergewicht<br>- Ehrenamtliche Weiterbildung<br>- Schaffung eines kultursensiblen und niederschwelligen Angebots zur Erreichung von Eltern mit Migrationshintergrund in Kindertageseinrichtungen in den Bereichen Ernährung und Bewegung<br>c) „Bärenstark – Ernährungs- und Bewegungsangebot für Eltern mit Kindern unter drei Jahren“	- Viele Kooperationen von Sportvereinen mit Kindertagesstätten und Schulen blieben bestehen<br>- Internetseite DoKi.Dortmund.de sammelt weiterhin Veranstaltungen zu Kindergesundheit sowie Tipps für Bewegungsstunden und gesunde Ernährung<br>- mehr als 8.000 Kinder, 3.000 Eltern und beinahe 500 Fachkräfte erreicht<br>- Einrichtungen für Kinder sind häufig durch eine Vielzahl von Projektanfragen überfordert<br>Familienbüros:<br>- sind Funktion als Service- und Anlaufstelle für Familien in vollem Umfang gerecht geworden<br>- zu Beginn gab es wenige Maßnahmen, Familienbüros konnten im Laufe des Projekts etliche Maßnahmen und Aktionstage zu initiieren bzw. gemeinsam mit Kooperationspartnern umsetzen Zahl der möglichen Kooperationspartner zum Thema Ernährung in Dortmund sehr eingeschränkt<br>- Maßnahmen im Projektzeitraum meist kostenfrei gleichbleibende Partizipation nach Projektende, wenn Kosten erhoben werden?<br>- Wichtig: wohnortnahes und passgenaues Angebot für junge Familien und Auswahl von für die Zielgruppe geeigneter Referent/-innen<br>- Bildungsnahe Schichten nehmen Informations- und Bewegungsangebot gerne an und verfügen aber bereits über Kenntnisse<br>- Schwer erreichbare Familien: formulieren eher keinen Bedarf und nehmen eher nicht an Angeboten teil<br><br>Elternbegleiter/-innen:<br>- mangelhafte Partizipation nicht aufgrund mangelnden Interesses, sondern Überwindung kultureller und sprachlicher Hürden oder Unkenntnis	auf die Prävention von Übergewicht (s. 2.3)	er-kinder-besser-essen-mehr-bewegen-doki/

			- Bewegungsangebot für Kinder, parallel dazu bereiten Eltern ein gemeinsames Mittagessen vor, das die Kriterien saisonal, kindgerecht und kostengünstig erfüllt<br>-Mitarbeiter/-innen arbeiten situationsorientiert, inhaltliche Angebote orientieren sich an den Fragen und Themen, die sich aus den Gesprächen mit Teilnehmer/-innen ergeben<br>d) „Partnerschaften mit Sportvereinen" zur frühzeitigen Anbindung und zur Schaffung von zusätzlichen Bewegungsangeboten für Kinder in Kitas<br>- Schaffung zusätzlicher Bewegungsmöglichkeiten<br>- Sensibilisierung von Kitas für das Thema Bewegung<br>- Kita und Verein verpflichteten sich in einem Vertrag eine bewegungsfreundliche Umgebung für Kinder zu gestalten<br>- eigenständige Gestaltung der Partnerschaft, StadtSportBund stand beratend zur Seite	über bestehende Strukturen oder Angebote der Einrichtungen<br>- Zeitmangel bei den Seminaren: einzelne Seminareinheiten mit Inhalten überfrachtet<br>- Unflexible Handhabung des Budgets: Projekt wuchs und wandelte sich in seiner Laufzeit, Verantwortliche konnten darauf nicht immer angemessen reagieren [illegible] Besser: einzelne Projektverantwortliche erhalten Verantwortung für ein Gesamtbudget innerhalb dessen Grenzen sie autonom über die Mittel verfügen können<br>- Ehrenamtlichkeit: Schwierigkeiten bei Verbindlichkeit und Absprache [illegible] Besser: Aufwandsentschädigung für durchgeführte Angebote könnte größere Verbindlichkeit in Bezug auf die Häufigkeit der durchgeführten Aktivitäten in Eirichtung als auch die Dokumentation und Planung herstellen<br>- Kinderbetreuung während der Qualifizierung ist wichtiger Faktor für Teilnahme<br>- Thematisch breitgefächerte Qualifizierung half dabei das Interesse potenzieller Elternbegleiter/-innen zu wecken und unterstütze die Kindertageseinrichtungen dabei an bereits bestehende Aktivitäten anzuknüpfen oder diese zu ergänzen<br>- Kennenlernen als eigenes Modul der Fortbildung: sehr offener Umgang untereinander, Ressourcenorientierung, Schaffung einer angenehmen Arbeitsatmosphäre, die auf gegenseitigem Respekt und Vertrauen beruht<br>- Elternbegleiter/-innen als Tandems in den Einrichtungen sind förderlich (Unterstützung und Motivation)<br>- Gewinnung der Elternbegleiter/-innen über Kindertageseinrichtungen: Persönliche Ansprache durch Erzieher/-innen waren förderlich		

				Bärenstark:<br>- Gruppe wuchs in kurzer Zeit durch Mund-zu-Mund-Propaganda, so dass auf Werbung verzichtet wurde eingespartes Geld konnte für zusätzliche Gruppe gespart werden (zweite Gruppe hatte aber nicht den Effekt, dass Gruppe sich teilt, sondern Eltern an beiden Tagen kommen Gruppen häufig überfüllt)<br>- Interesse der Teilnehmer/-innen für gesunde Ernährung konnte aufgegriffen und gezielt erweitert werden<br>- visuell ansprechende und erlebnisorientierte Aktionen besonders beliebt und erfolgreich, z.B. selbst gestaltete Ernährungspyramide, Zuckerwürfel in Getränken<br>- Theorie nicht für alle interessant. Manche kamen nur wegen der kostenlosen Mahlzeit<br>- Eltern überrascht, dass Kinder häufig mehr Lebensmittel akzeptieren, als sie dachten<br>- Schwieriger Transfer der Bewegung in den Alltag umlernen der Eltern ist mit Unannehmlichkeiten verbunden, z.B. draußen spielen auch bei eher schlechtem Wetter<br>- durch Beengte Wohnverhältnisse können viele Bewegungsanregungen zu Hause nicht umgesetzt werden Verhaltensauffälligkeiten konnten beobachtet werden<br>- stundenweise Betreuung für Kinder wäre sinnvolle Ausbaumöglichkeit<br>- persönlicher Kontakt und persönliche Begleitung durch den Eltern bekannte Betreuer/-innen machen die Maßnahme so erfolgreich<br>- Im Vorhinein festgelegter Rahmen der Maßnahme hindert bedarfsgerechte Weiterentwicklung, z.B. wurde der Antrag der Eltern eine/-n Kinderarzt/ärztin einzubeziehen abgelehnt Lösung: pensionierter Kinderarzt und Kinder- und		

				Jugendpsychiater konnte für freiwillige Mitarbeit gewonnen werden<br>- Hemmender Faktor: Kosten es hat sich gezeigt, dass viele Eltern selbst bei sehr geringen Kosten der Maßnahme fernbleiben; Lösung: Eltern übernehmen im Rahmen ihrer individuellen Möglichkeiten Aufgaben für die Gesamtgruppe wie z.B. Handtücher waschen oder Lebensmittel von zu Hause mitbringen<br>- Einsatz von Fragebögen zur Evaluation irrelevant: Eltern, die nur sporadisch kamen, konnten nicht in Auswertung einbezogen werden; bei Überfüllten Gruppen häufig keine Zeit zur Befragung; bei ausgefüllten Fragebögen wurden Fragen häufig in Richtung vermuteter Erwünschtheit bearbeitet<br>Partnerschaft mit Sportverein:<br>- Ausschreibung des StadtSportBundes an Sportvereine und Kitas erziele Rücklauf von knapp 10%; über persönliche Ansprache von Sportvereinen konnte in 90% der Gespräche der Sportverein von Teilnahme überzeugt werden höhere Personalressource notwendig<br>- einige Sportvereine betreuten mehrere Kitas<br>- Trotz Kontaktherstellung kamen einige Partnerschaften nicht zu Stande eigenständige Entwicklung einer Partnerschaft funktionierte nicht immer<br>- Nutzen für Kitas: Schaffung von mehr Bewegungsräumen und -gelegenheiten für Kinder<br>- Nutzen für Sportvereine: Überarbeitung von Inhalten und Angeboten, um Kinder zu erreichen, die sonst keinen Zugang zu Sportvereinen finden<br>- Umsetzbarkeit der Projekte war abhängig von Kapazitäten der Sportvereine und dessen Übungsleitern<br>- Vereine bringen häufig starre Strukturen mit, die Zusammenarbeit mit Kita erschweren können		

				- personelle Ressourcen für Koordination und Begleitung der Partnerschaften wurden nicht ausreichend eingeplant		
Gesundheitsteams vor Ort – Mainz-Neustadt und Trier-Nord	Seit 2006	Caritasverband Mainz e. V.	- niederschwellige und nachhaltige Gesundheitsangebote im Wohngebiet durch Gesundheitsteams (stadtteilbezogene Akteur/-innen aus psychosozialen und pädagogischen Bereichen, die eng vernetzt in einer Arbeitsgruppe zusammenarbeiten Koordination über Steuerungsgruppe<br>- bei der Planung von Maßnahmen wird der Aspekt der Nachhaltigkeit besonders berücksichtigt<br>- besondere Ansprache von Familien, aber auch Alleinstehende und ältere Bürger/-innen in sozial benachteiligten Wohnquartieren<br>a) „Stark durch Bewegung" Bewegungs- und Haltungsorientierte Angebote für schwangere Frauen oder Frauen, die gerade entbunden haben<br>b) „Gesundheitsberatung, Information und Austausch: Frühstück mit Müttern und Babys" niederschwelliges Angebot für Mütter, sich beim gemeinsamen Frühstück mit Expert/-innen auszutauschen und zu informieren<br>c) „Sprechstunde vor Ort" niederschwelliges allgemeinmedizinisches und zahnmedizinisches Beratungsangebot „Türkische Frauen in Bewegung" Körper-, bewegungs-, haltungs- und gesundheitsorientierte Angebote für türkisch sprechende Frauen<br>- Turnus: wöchentlich eine Stunde<br>d) „Offener Gesprächskreis Frauengesundheit" niederschwelliges offenes Angebot als gruppenpädagogisches Angebot zu Fragen der Frauengesundheit in türkischer Sprache mit der Möglichkeit von Einzelgesprächen<br>e) „Medizinstudierende Dolmetschen" (MSD) Aufbau eines Dolmetscherdienstes	Um Zielgruppe besser zu erreichen, bedarf es:<br>- alternative Zugangsmöglichkeiten (z.B. typische Treffpunkte und Versammlungsorte, religiöse Zentren, Familien) geeignet sind z.B. bereits bestehende Begegnungsräume (kaffee- oder Teestube, Stadtteiltreffs, Gemeinderaum, Spielplätze und sonstige öffentliche Plätze) in den zu bestimmten Zeiten Ansprechpartner/-innen anwesend sind (z.B. im Rahmen einer Sprechstunde)<br>- niederschwellige, aktiv aufsuchende Maßnahmen<br>- Wohnortnahes Angebot<br>- Maßnahmen, die möglichst dicht in den Alltag integriert sind und mit herkömmlichen Angebotsstrukturen des Gesundheitssystems verknüpft werden		http://www.wogebe.de/sozialeprojekte/gesundheitsteams/Konzept-GesundheitsteamsvorOrt-2007.pdf

			von Medizinstudierenden, um Migrant/-innen zu Arztbesuchen zu begleiten<br>-Dienst auf Abruf<br>f) „Sport und Gesundheit für aktive Migrantinnen“ Sport- und Gesundheitsangebot für Migrantinnen, die schon einem anderen Projekt der Gesundheitsteams teilgenommen haben (= Verbesserung der Nachhaltigkeit)<br>g) „Stark durch Stillen: Vorteile des Stillens – Was ist zu beachten?“ – Unterstützung durch Informationen und Beratung“ sozialraumorientiertes Angebot von Stillberaterinnen<br>h) „Migranten für Migranten (MiMi) Interkulturelle Gesundheit in Deutschland“ Gesundheitsmediator/-innen ausländischer Herkunft führen mehrsprachige und kultursensible Informationsveranstaltungen zu gesundheitsbezogenen Themen durch<br>i) „Frauen in Bewegung“ Durchführung gesundheitswirksamer Bewegungsangebote			
Kinder.leicht. gesund e. V. – Bremer Plattform für Ernährung und Bewegung	2007 bis 2009	Stadt Bremen, gefördert durch das Bundesministerium für Ernährung, Landwirtschaft und Verbraucherschutz	- Motivation und Schulung von Multiplikator/-innen<br>- Schaffung lokaler Netzwerke (Kitas, Schulen, Vereine und Familien) zur Gestaltung nachhaltiger Strukturen<br>- Aktive Einbindung von Eltern und Familien kennenlernen der Bedeutung von ausgewogener Ernährung und körperlicher Aktivität für die Gesundheit des Kindes<br>- besonderes Augenmerk liegt auf der Ansprache von sozial benachteiligten Personen und Migrant/-innen<br>- Aufteilung in 7 Leitprojekten mit 40 Einzelprojekten<br>a) „Vom Mutterbauch zur Ganztagsschule“ Zielgruppenworkshop für 0-3-Jährige: Grundlagenaustausch und Diskussion, wie Projekte für diese Zielgruppe attraktiver gemacht werden können (22.11.2007 und 11.07.2008)	Printprodukte:<br>- Überflutung mit Inhalten vermeiden, sollten lediglich als ergänzendes Medium genutzt werden<br>- sprachliche Gestaltung beachten<br>- Sichtweise und Blickwinkel der Zielgruppe beachten: „dick“ sein ist im arabischen Raum kein Problem, sondern Ausdruck von Wohlstand, folglich werden Flyer, die auf „zu dicke“ Kinder abzielen eher als nicht wichtig eingestuft<br>- Vermeidung des „pädagogischen Zeigefingers“<br>- Besser: Präsentation eines Beratungsangebots<br>- Auswahl geeigneter Fotos, mit der sich Zielgruppe identifizieren kann und die nicht diskriminieren (häufig mit erhöhtem Kostenaufwand verbunden<br>*„Entwicklung Multiplikator/-innenschulung für Hebammen“:*	Übergewicht:<br>- „Elternkurse“ strukturelle Schulung von Eltern übergewichtiger Kinder mit parallellaufendem Sport- und Bewegungsangebot	https://www.in-form.de/netzwerk/projekte/kinder-leicht-gesund-bremer-plattform-fuer-ernaehrung-und-bewegung/

			- „Entwicklung Multiplikator/-innenschulung für Hebammen“<br>- „Elternschulungen im Rahmen von Schwangerschaftskursen“: Entwicklung diverser Medien, um die Kursinhalte stadtweit im Rahmen normaler Schwangerschaftskurse personalunabhängig per DVD und Infobroschüren kommunizieren zu können<br>- „Tanzen und Tafeln“ = Tanz Projekt für Schwangere und junge Mütter mit besonderem Fokus auf sozial benachteiligten Schichten oder Migrantinnen<br>- „Kursleiterqualifizierung für Übungsleiter/-innen und Erzieher/-innen in Krabbelgruppen und Kitas“<br>b) „Wege und Räume beleben“<br>- „Bewegungs-Ernährungs-Mobil (Bemil) = PKW-Anhänger oder Lastenfahrrad, das Materialangebot für alle Altersgruppen zum Thema Ernährung bereithält [illegible] gezielte Ergänzung des Materialangebots auch für Klein- und Kleinstkinder<br>- „Geführte Familienfahrten zu Bauernmärkten und -höfen“<br>c) „Transfer in sozialbenachteiligte Stadtteile“	-Multiplikator/-innenschulungen: Heterogenität innerhalb Berufsgruppe beachten und in Schulung darauf eingehen → ggf. Zielgruppen genauer definieren und Schulungsinhalte darauf abstimmen<br>- Überlegung: Einbettung von Multiplikator/-innenmaßnahmen in berufliche Ausbildung<br>*„Elternschulungen im Rahmen von Schwangerschaftskursen“:*<br>- erstellte Materialien für erst in Rückbildungskursen einsetzen, da nur geringer Bezug der werdenden Eltern zu den Bedürfnissen des erwarteten Kindes<br>*„Tanz und Tafel“:*<br>- Teilnahme für türkische Frauen teilweise schwierig, da Männer intervenierten → Frauen würden durch Kurs zu selbstständig und entfernten sich zu weit von türkischer Kultur<br>- große Verunsicherung der Schwangeren und jungen Mütter im Thema Stillen und Säuglingsernährung → teilweise widersprüchlich Informationen von Pflegepersonal, Krankenhaus und Literatur<br>- Projekt läuft auch nach Abschluss des Programms unter anderem Träger weiter<br>*„Kursleiterqualifizierung für Übungsleiter/-innen und Erzieher/-innen in Krabbelgruppen und Kitas“:*<br>- Erzieher/-innen und Übungsleiter/-innen sind mit dem Thema Bewegung vertraut<br>- Interesse an Projekt kurzzeitig ausgeschöpft, Schulungen wurden dann aber nach einjähriger Pause wieder angeboten<br>*„Bewegungs-Ernährungs-Mobil (Bemil)“:*<br>-Projekt läuft auch nach Abschluss des Programms aufgrund der großen Nachfrage weiter (zweiter Anhänger wurde in Auftrag gegeben)<br>- Teilnehmenden-Akquise hatte sich als besondere Herausforderung erwiesen, da		

				klassische Werbung mit Printprodukten bei der Zielgruppe nur eingeschränkt wahrgenommen wurde →Besser: persönliche Ansprache durch eine vertraute Person (persönliche Ansprache = sehr zeit- und ressourcenintensiv)<br>- klassische Kursangebote mit Startdatum, fester Teilnehmerzahl und fixen Terminen wir nur zögerlich wahrgenommen und wirkt auf Zielgruppe abschreckend → besser: Projekte mit offener Form und unverbindlichem Zugang<br>- Projektkosten sollten sich an Ressourcen der Zielgruppe orientieren: geringe Beiträge z.B. als Umlagen für Lebensmittel können die Wertigkeit des Projekts sogar verbessern<br>- Örtliche Verankerung ist wichtig: kurze Wege zum Veranstaltungsort (häufig eingeschränkte Mobilität der Zielgruppe beachten)<br>*Finanzen:*<br>- Finanzplan bei Projektantrag im Herbst 2006 bis 2009 verbindlich fest zu schreiben, war vollkommen praxisfremd und barg für Projektpartner erhebliche Risiken<br>- formale haushaltstechnische Anforderungen an das Projekt: Detaillierte Finanzplan musste im Herbst 2006 schon stehen, aktuellen Anpassungsbedürfnissen konnte nicht nachgekommen werden<br>- verbindliche Festlegung zur Erbringung von Drittmitteln schon 2006 definiert → widerspricht tatsächlichen Gegebenheiten in der Praxis<br>- Mittel auf Landesebene sind angesichts der sehr knappen Ressourcen nur sehr schwer erhältlich, schon gar nicht mit Garantie über mehr als drei Jahre<br>- Förderung auf Stadtteilebene wird nur im laufenden Haushaltsjahr gewährt (Beiräte wissen nicht, welches Budget sie im Folgejahr zur Verfügung haben)		

				- Flächendeckende Umsetzung nicht möglich, da Personal und Mittel fehlen<br>- Besser: Projektgelder sollten zukünftig komplett oder anteilig in Budgetform zur Verfügung gestellt werden, um kurzfristig und flexibel handeln zu können<br>- angemessene Vergütung für Evaluation sollte in zukünftigen Projekten berücksichtigt werden<br>- Bürokratischer Aufwand durch Dokumentation der Projekte muss zusätzlich erbracht werden und ist nicht im kalkulierbar<br>- schlechte personelle Ausstattung und fehlende Ressource (z.B. Zeit oder Räumlichkeiten) erschwert Durchführung der Projekte<br>- Zugehörigkeit der einzelnen Projekte zum Netzwerk war in der Presse nicht immer ersichtlich → in der Zukunft sollten Projektpartner zu einheitlicher Außendarstellung verpflichtet werden<br>- besser schon bestehende Projekte mit hoher Partizipation fördern als immer wieder neue Projekte anzuschieben, bei denen im Grunde von Beginn an schon klar ist, dass die nach Ende der Förderphase auslaufen werden		
Potpourri	Januar 2015	Frecher Spatz - FaZ (Familienaktiv-Zentrum)	- Betreuungsprogramm für Eltern und Kinder an fünf Tagen die Woche mit täglichen Angebotsmöglichkeiten<br><br>Montag:<br>- Teilnehmer/-innen werden zu Außenterminen begleitet<br>- Hilfe bei der Ausfüllung von Formularen<br>- Hilfe bei Terminvereinbarungen<br>- Gespräche mit Jugendamt<br>- etc.<br>Dienstag:<br>- Sprachförderung<br>- geeignet für alle Teilnehmer/-innen (sowohl Teilnehmer/-innen mit guten Sprachkenntnissen, also auch Teilnehmer/-innen mit keinen bis wenig Deutschkenntnissen	- Entwicklung und Förderung von interkultureller Kommunikation<br>- Entwicklung und Förderung des Spracherwerbs<br>- Förderung der Gesundheit der Kinder und der ganzen Familie<br>- Vermittlung von Fachwissen<br>- Aufbau der eigenen Rezeptsammlung<br>- Erkennen und Umgang mit eigenen Stärken und Schwächen<br>- Entwicklung und Förderung von Geruchs- und Geschmackssinn<br>- Kennenlernen verschiedenster frischer Nahrungsmittel, Kräuter und Gewürze<br>- Erweiterung des häuslichen Speiseplans<br>- Motivation, Animation und Anleitung zum Nachmachen und Sammeln	EP-A.: Entwicklung und Förderung von Geruchs- und Geschmackssinn	http://www.frecherspatz.de/images/Zwischenbericht_Modellprojekt_Potpourri.pdf

			Mittwoch:<br>- „Bewegungs-Picknick“<br>- gezieltes Bewegungsangebot und Austausch in der Gruppe<br>Donnerstag: Gemeinsames Kochen und Hauswirtschaften<br>- Sensibilisierung für eine ausgewogene Ernährung für die ganze Familie und einen bewussten Umgang mit Lebensmitteln<br>Freitag: Ausflüge und Exkursionen<br>- Spielplätzen und Parks<br>- Tiergarten<br>- Bücherei<br>- Geschäfte<br>- Kitas<br>- Naturkundemuseum<br>- Spreeufer<br>- Bus und Bahn fahren<br>- Schwimmbad<br>- Minigolf<br>- u.v.m.<br>- Elterncafé: kostenfrei und ohne Anmeldung zum Austausch plus Angebot von Getränken und Essen (Snacks, Obst, Gemüse)	- Überwinden der „Scheu“ mal was anderes auszuprobieren<br>- TeilnehmerInnen übernehmen Verantwortung innerhalb der „Potpourri Gemeinschaft“<br>- Pflege und Förderung von Tradition (Rezepte und Zubereitung gestern und heute)<br>- Anerkennung und Spaß<br>- Gemeinschaft wird aktiv gelebt<br>- Wertevermittlung (Respekt gegenüber Menschen und Lebensmitteln, Gemeinsinn, Wertschätzung, sich Zeit nehmen)		
Kommunal-verankerte Projekte mit der Zielgruppe: Multiplikator:innen						
9+12 Gemeinsam gesund in Schwangerschaft und erstem Lebensjahr	05/2011 - 09/2014	In Form – Deutschlands Initiative für gesunde Ernährung und mehr Bewegung und peb – plattform ernährung und bewegung e. V.	- Erweiterung der gesetzlich vorgesehenen Vorsorgeuntersuchungen in der Schwangerschaft und im Säuglingsalter, welche vornehmlich der Krankheitsfrüherkennung dienen, um präventive Elemente systematische Beratung zu den Themen Ernährung und Bewegung<br>- Frauenärzt/-innen, Hebammen sowie Kinder- und Jugendärzt/-innen verweisen bei schwerwiegenden Ernährungsstörungen auf das Beratungsangebot bei den Ernährungsberater/-innen hin<br>- (werdende) Eltern erhalten zusätzliche Informationen zu bedarfsgerechten und konkreten Angeboten der Gesundheitsförderung im Landkreis Ludwigsburg	- Mangelnde Bewegung wirkt sich negativ auf die Schwangerschaft aus, egal ob die Frau normalgewichtig oder übergewichtig ist<br>- standardisierte Beratung zu Ernährung und Bewegung ermöglicht frühzeitige Feststellung von Fehl-, Unter- und Überernährung sowie eines Bewegungsmangels<br>- Projekt erreichte Familien aller Bildungsniveaus (in Modellregion Ludwigsburg nahmen überdurchschnittliche viele adipöse Frauen oder Frauen mit Migrationshintergrund am Projekt teil)<br>- Schwangere achteten verstärkt auf feste Abläufe und das Einnehmen der Hauptmahlzeiten<br>- zum Ende des 2. Lebensmonats wurden 70 % der Säuglinge voll und 16 %	Übergewicht:<br>- Ansprache sowohl normalgewichtiger als auch übergewichtiger Frauen in der Schwangerschaft Primär- UND Sekundärprävention	https://www.in-form.de/netzwerk/projekte/9-12-gemeinsam-gesund-in-schwangerschaft-und-erstem-lebensjahr/

			- Liste mit örtlichen Sportangeboten, Ernährungs- und Laktationsberatungen und deren Kursangebote werden an die Eltern verteilt<br>- Erstellung von acht Filmsequenzen, die zum jeweiligen Untersuchungszeitpunkt passende Botschaften vermitteln (= Ergänzung zum persönlichen Gespräch) Filmmaterial dient nur der Vertiefung und ersetzt NICHT das Beratungsgespräch: Ziel: (werdende) Väter emotional ansprechen<br>*10 Untersuchungszeitpunkte:*<br>- vier Vorsorgeuntersuchungen beim Gynäkologen erweitert um eine präventive Beratung zu jeder Untersuchung<br>- zwei Interventionszeitpunkte für Hebammen (kurz vor u. nach der Entbindung)<br>- vier weitere Interventionszeitpunkte für Kinder- und Jugendärzte nach der Geburt innerhalb der ersten 12 Monate des Kindes<br>- Präventionspass und Präventions-Checklisten unterstützen das Beratungsgespräch und dokumentieren den Beratungsverlauf<br>- Kurzfilme mit den Kernbotschaften ergänzen das Beratungsgespräch und sprechen insbesondere Väter als Zielgruppe an<br>*Ziel:*<br>Reduktion und Vermeidung von kindlichem Übergewicht und Adipositas bei Mutter und Kind, Bessere Versorgungsstruktur für junge Familien, Verbesserung existierender Angebote speziell für die Zielgruppen der Schwangeren (Sportvereine, Ernährungsberatung, etc.)	teilweise gestillt; bis Ende des 4. Lebensmonats wurden 62 % der Kinder voll und 112 % teilweise gestillt<br>- Empfehlungen zur Beikost oder Einführung der Familienkost wird in hohem Maße umgesetzt<br>- Projektteilnehmerinnen halten in der Schwangerschaft und nach der Geburt mehrheitlich an die Empfehlungen zum Genussmittelkonsum (Schwangerschaft: 90 % rauchen nicht oder nicht mehr, 8,5 % rauchen weniger; Nach der Schwangerschaft: 95 % rauchen nicht)<br>- über 90 % der Schwangeren jungen Mütter trinken keinen Alkohol<br>- geringere Kaiserschnittrate als im Bundes- und Ländervergleich<br>- Schwangere steigerten sowohl Alltagsbewegung als auch bewusste körperliche Aktivität im Gegensatz zu Frauen einer bundesweiten Kontrollgruppe Vor allem Frauen mit erhöhtem Präventionsbedarf wurden damit zur erstmaligen Aufnahme sportlicher Aktivitäten motiviert (aktiver Lebensstil wird häufig auch nach der Geburt beibehalten)<br>- nur 12 % der Neugeborenen weisen erhöhtes Geburtsgewicht auf (Kontrollgruppe: 19 %) Projektteilnahme wirkt sich positiv auf Geburtsgewicht der Kinder aus<br>- Maßnahmen unterstützen den Grundsatz einer umfassenden und widerspruchsfreien Aufklärung und Beratung zu Ernährungs- und Bewegungsthemen, um späteres Übergewicht bei den Kindern zu vermeiden<br>- Kommunikation und Vernetzung der Multiplikator/-innen untereinander wurde durch das Projekt verbessert<br>- Informationsmaterialien und -medien können Programm gut ergänzen		

BILDUNGSORT ESSTISCH!	2 Termine - 20.10.2020 und Frühjahr 2021	Verbraucherzentrale Hessen e. V. Fachgruppe Lebensmittel und Ernährung, gefördert durch Hessisches Ministerium für Soziales und Integration	Seit 2007 6 Workshops zum Thema Ernährung von Kindern, davon 3 mit Schwerpunkt Ernährungsbildung<br>a) "Mit Kindern Essen" – Erziehung und -beziehung im Kleinkindalter<br>- praxisorientierte Anleitung für Pädagog/-innen<br>- die Welt der Lebensmittel mit allen Sinnen entdecken<br>b) „Ausgewogene Ernährung im Kindesalter“<br>- Beantwortung von gängigen Fragen bezüglich einer gesundheitsfördernden Ernährung<br>c) „Essen mit allen Sinnen“ und mehr…: Ernährungsbildung im Kleinkindalter		EP-A.: pädagogischer Ansatz	https://www.verbraucherzentrale-hessen.de/lebensmittel/bildungsort-esstisch-fachtag-fuer-fachkraefte-und-interessierte-24955
„Ganztags leicht(er) leben“	2006 bis 2009; 2009 bis 2010	Beschäftigungs- und Qualifizierungs-gesellschaft Ostholstein gGmbH, Eutin	a) Ausbildung von 12 Familienassistent/-innen in ernährungs- und sozialpädagogischen Inhalten, Aspekten des Rechts, der Kommunikation, der Psychologie<br>- Besuche durch Familienassistent/-innen bei den Familien zu Hause<br>- Familienassistentinnen bieten offene Frühstücks- und Sprechstunden in Institutionen an<br>- Inhalte der Beratung: Ernährung und Bewegung, psychosoziale und praktische Unterstützung der Eltern bei verschiedenen Problemlagen wie Trennung, schulischen Problemen, insbesondere aber Erziehungsfragen	- Beratung und die individuelle Begleitung durch Familienassistent/-innen, die Kombination aus Erziehungsberatung und dauerhafter Ernährungsbildung für die Eltern einerseits und verbesserten Bewegungsangeboten für die Kinder andererseits zeigte sich als sehr wirkungsvoll und sorgte dafür, dass viele Familien Schritt für Schritt zu mehr Gesundheit und sozialer Lebensqualität gekommen sind<br>- Netzwerke nur noch teilweise aktiv, keine Erstellung einer Geschäftsstellung gelungen<br>- Erfolg: viele Kitas (und Schulen) haben Projektinhalte in den Alltag übernommen, Stabilisierung der Arbeit der Familienassistent/-innen durch Fortsetzung und Weiterbildung ihrer Arbeit<br>- Akquirieren zusätzliche Mittel nötig, um die Arbeit des Netzwerkes fortzusetzen<br>a) Familienassistentinnen:<br>- Starke Abweichung zwischen Planung und Umsetzung: große Hürde bei der Vermittlung von „Einsätzen“ der Familienassistentinnen bei betroffenen Familien, obwohl Bedarf besteht; Familien nahmen Angebot häufig als Einmischen in Privatangelegenheiten	EP-A.: die Familien-assistent/-innen wurden unter anderem im Punkt der Psychologie geschult	https://www.in-form.de/netzwerk/projekte/ganztags-leichterleben/

				wahr Kontinuität, Verlässlichkeit, persönlich Ansprache und langer Atem notwendig, um Netzwerk und Akzeptanz auszubauen		
Gesund sind wir stark! – Sağlıklı daha güçlüyüz	2006 bis 2009; 2009 bis 2010	ZAGG (Zentrum für angewandte Gesundheitsförderung und Gesundheitswissenschaften GmbH), Plan- und Leitstelle Gesundheit in Friedrichshain/Kreuzberg, SW ernährungswissenschaftliche Dienstleistungen	- Unter Einbeziehung der besonderen Stärken des Projektbezirks (kulturelle Vielfalt, nachbarschaftliche Kiezstruktur, reiche Projektlandschaft, lange Tradition der Bürger/-innen-Beteiligung) entstand ein umfangreiches Schulungskonzept für Multiplikator/-innen<br>a) Weiterbildung als „Gesundheitstrainer/-in" vor allem von (möglichst) muttersprachlichen Multiplikator/-innen aus sozialen Einrichtungen im Bezirk, die beruflich mit Menschen mit Migrationshintergrund in Kontakt kommen (z. B. Hebammen)<br>b) Weiterbildung als „Gesundheitsmentor/-in" von muttersprachlichen Laien, die im privaten Umfeld<br>c) Aufbau einer festen Koordinationsstelle, die für Anfragen zur Verfügung steht und die Gesundheitstrainer/-innen und -mentor/-innen für Vorträge und Beratungsangebote zu den Themen gesunde Ernährung und mehr Bewegung vermittelt<br>- Mitwirken bei der bezirklichen Gesundheitsplanung<br>- Verbesserung der Kooperation zwischen allen Einrichtungen und Personen, die mit Schwangeren und jungen Familien arbeiten<br>- Stärkung der Elternkompetenz<br>- Identifizierung von Versorgungslücken Initiierung von Notwendigen Maßnahmen und Projekten<br>- Vermeidung von Doppelangeboten<br>*Ablauf der Maßnahmen:*<br>- Vorbereitungsphase: Erarbeitung des kultursensiblen Lehrgangscurriculums (Projektpartner zusammen mit Dozent/-innen und muttersprachlichem Expert/-innen)	- im Zeitraum 2006 bis 2008 konnten 56 Multiplikator/-innen ausgebildet und zertifiziert werden, davon 44 als Trainer/-innen und zwölf als Mentor/-innen<br>- Gesundheitstrainer/-innen haben mit ihrem Zusatzwissen in ihrem beruflichen Umfeld Hunderte von Familien erreicht<br>- Veränderungen im Ernährungs- und Bewegungsverhalten konnten angestoßen werden (z. B. Reduzierung von Süßigkeiten und süßen Getränken, mehr Obstverzehr, häufigere Besuche auf dem Spielplatz).<br>- einige Gesundheitsmentor/-innen sind auf Honorarbasis weiterhin aktiv und organisieren z. B. Bewegungsangebote in Stadtteilhäusern (Winterspielplatz, Familiendisco)<br>- Seit November 2010 gibt es die Planungs- und Koordinierungsstelle Gesundheit als (vorläufig) institutionalisiertes Angebot im Bezirksamt Friedrichshain-Kreuzberg Schaffung guter Voraussetzungen für Verstetigung des Projekts<br>- 2009 mit der "Good Practice" Urkunde ausgezeichnet<br><br>- Häufiges Umbesetzen der Projektkoordination befristete halbe Stelle, Koordinator/-innen verließen das Projekt, als unbefristete Vollzeitstelle angeboten wurde<br>- Umzug Koordinationsstelle: neue räumliche Situation so beengt, dass kein Platz für neues Projektbüro war; lediglich stundenweises Arbeiten möglich erheblicher Mehraufwand<br>- erstellte Flyer werden sehr gut angenommen<br>*Trainer/-innen und Mentor/-innen:*		https://www.in-form.de/netzwerk/projekte/gesund-sind-wir-stark-saglikli-daha-gueclueyuez/

			- Lehrgangsphase: Akquise der Gesundheitstrainer/-innen und Gesundheitsmentor/-innen und deren Ausbildung<br>- Praxisphase: Integrieren des erworbenen Wissens in die Beratung und Begleitung der Zielgruppe<br>*Ziel:* Vermittlung von gesunder Ernährung und mehr Bewegung in jungen Familien mit Migrationshintergrund „auf Augenhöhe“ Themen werden in vorhandene kulturelle Ressourcen und Werte der Familie eingebettet	- Begleitung der Trainer/-innen und Mentor/-innen durch Projektteam durch regelmäßige Supervision und Qualitätszirkel Qualität, Effektivität und Nachhaltigkeit der Beratungsarbeit wird sichergestellt, Arbeit wird transparent gemacht, möglicher Veränderungsbedarf wird aufgezeigt<br>- Trainer/-innen und Mentor/-innen hatten unterschiedliches Verständnis von Beratung: Mentor/-innen seltener klassische Beratungen mit einbestellen und Gespräch große Vielfalt an Beratung, Informationen können in unterschiedlichster Form zielgruppengerecht transportiert werden<br>- Unterschiedlicher Beratungskontext und Sprachgebrauch: Trainer/-innen beraten eher institutionell und im beruflichen Zusammenhang und sprachen dabei überwiegend deutsch, Trainer/-innen mit Migrationshintergrund beraten auch in ihrer Muttersprache; Mentor/-innen beraten auch im privaten Zusammenhang, z.B. Freunde und Familie, häufig in Muttersprache<br>- Art der Beratung: Trainer/-innen überwiegend theoretische Vermittlung von Inhalten, Mentor/-innen häufiger praktische Anregungen, wenden erworbenes Wissen in der eigenen Familie aktiv an<br>- Themenbereiche beider Gruppen: Ernährungsfragen, Veränderungen durch Schwangerschaft/ Stillzeit, Bewegung<br>- Trainer/-innen konnten nur schwer zur Teilnahme an den Supervisonstreffen und Qualitätszirkeln motiviert werden, weil Verbindlichkeit der Teilnahme an diesen Treffen am Anfang des Projekts nicht ausreichend kommuniziert wurde Besser: neu ausgebildete Trainer müssen an vier von sieben Supervisionstreffen und Qualitätszirkeln verpflichtend teilnehmen, um Abschlusszetrifikat zur erhalten		

				- Mentor/-innen konnten nur schwer zur Teilnahme an den Supervisonstreffen und Qualitätszirkeln motiviert werden supervisorische Begleitung wurde eingestellt und durch ein intensives, aufsuchendes Betreuungsangebot ersetzt (Diplom-Pädagogin berät Mentor/-innen in Einzelgesprächen)<br>- Coachingangebot für Trainer/-innen und Mentor/-innen ¾ nahmen das Angebot an, die Meisten Fragen konnten aber während der Supervisionen und Qualitätszirkel geklärt werden<br>- Rückmeldungen der Lehrgangsteilnehmer/-innen, dass Bedarf zu Themen besteht, die im Lehrgang behandelt werden Veränderung der bestehenden Materialien und Aufnahme zusätzlicher Materialien ins Beratungshandbuch und Organisation zusätzlicher Fachlicher Weiterbildung zu den Themen: Umgang mit Allergien, Schutz vor Unfällen im Haushalt (kindgerechtes Wohnen, Sicherheit) und Folgen von Adipositas<br>*Evaluation durch ZIWI-Methode:*<br>„Methode der Entwicklung lokaler Ziele und Wirkungswege, ZIWI-Methode“<br>- Erfahrungen des Projektteams, dessen Mitglieder auf unterschiedliche Weise zu den Gesundheitstrainer/-innen und -mentor/-innen in Kontakt standen, werden zusammengeführt. Im Rahmen des Modellprojekts können dann entsprechende Anpassungen des Konzepts auf die erarbeiteten Wirkungswege vorgenommen werden<br>- Herausarbeiten der Wirkungswege, die die Intervention und das Gesamtziel des Projekts miteinander verbinden Definition von Meilensteinen, Indikatoren, mit deren Hilfe das Erreichen des jeweiligen Meilensteins überprüft werden kann, sowie durch die Festlegung geeigneter Evaluations- und		

				Dokumentationsmethoden, mit denen Indikatoren gemessen werden können<br>*Einrichtung einer Projekteigenen Homepage:*<br>- Sichtbarkeit des Projekts für interessierte Institutionen und Einrichtungen konnte so verbessert werden<br>-projektbeteiligte Migrant/-innen schätzen Internetpräsenz als weniger nützlich ein<br>*Hemmend:*<br>- sind Verhaltensweisen von Eltern und Kindern bereits etabliert, fällt eine Verhaltensmodifikation schwer (z.B. Verhaltensänderung bei übergewichtigen Erwachsenen)		
„Kinderleicht-Quartier - stärken. Was wirkt!"	2006-2009	Verein Gesundheitshaus Gelsenkirchen e. V.	- Ziel des Projekts: weniger Kinder mit Übergewicht bei der Einschulungsuntersuchung<br>a) Babybrei-Kurse<br>b) Kochen für die Kleinsten<br>c) Kochkurse für Väter<br>d) Schulung des Teams Familienbildung-Familienförderung<br>e) Stillkampagne & Label Babyfreundlich<br>f) „Hem lezzet ve saglik“ – praxisorientierte Ernährungsschulung für türkische Familien: Vermittlung der Botschaft „Auch typisch türkische Gerichte lassen sich fettarm und gesund zubereiten“<br>g) „KINDELEICHT-Talk“<br>- Aufbauen von Handlungskompetenz in Bezug auf gesunde Ernährung und Bewegung der schwer erreichbaren Zielgruppe<br>- (Laien)-Moderator/-innen wurden geschult und ginge gezielt auf Eltern zu und animierten diese, andere Eltern aus ihrem Bekanntenkreis zu einer thematischen Gesprächsrunde und der gemeinsamen Zubereitung einer gesunden Mahlzeit einzuladen	- einzelne Projekte brauchten mehr Zeit und Geld<br>- nicht alle Ziele konnten erreicht werden, andere wurden stark verändert<br>- einzelne Kurse fielen wegen geringer Nachfrage aus<br>- Kooperationspartner/-innen sind abgesprungen<br>- die starre Gliederung in Entwicklungs-, Etablierungs-, Stabilisierungs- und Nachbereitungsphase war für auftretende Veränderungen nicht sinnvoll beizubehalten und wurde dementsprechend angepasst<br>- insgesamt wurde das Projekt sehr positiv aufgenommen - neben Schulen und Kindertagesstätten engagierten sich Vereine und Eltern<br>- dank der guten Dokumentation lassen sich die Konzepte auch auf andere Städte mit ähnlichen Strukturen übertragen<br>*„KINDELEICHT-Talk“:*<br>- sehr hohe Nachfrage nach Veranstaltungen<br>- Ansprache auf Augenhöhe<br>- Gäste des Talks äußerten, dass Sie gerne mehr Input auch von Expert/-innen erhalten möchten Expert/-innen wurden hinzugezogen		https://www.in-form.de/netzwerk/projekte/kinderleicht-quartier-staerken-was-wirkt/

				- Weiterentwicklung des Formats: zusätzliches gemeinsames Kochen „Talk and Cook“ (Kochen und Essen nach der Ernährungspyramide, Gut und preiswert kochen, Kochen zur Verhinderung von Übergewicht, Kochen für Kleinkinder)<br>- Maßnahmen vor allem in den Familien erfolgreich, die sonst nur schwer zu erreichen sind		
„Macht euch auf die Socken“	2006 bis 2009	Kreis Herford - Der Landrat - Gesundheit	- Hebammenschulung<br>- Kindertagesstätten-Ernährungskurse<br>- Angebote für Migrant/-innen: Gesundheitswochenende „Fit und müde und rund“ und Mütter-Schwimmkurse<br>- geschulte Familienbesucher/-innen und Elterntreffs schafften eine gute Basis für weitere Gesprächskreise<br>- „Kinderärzte empfehlen“ - Beratungsleitfäden für die U-Untersuchungen (altersspezifische Informationen zu den Themen Ernährung und Bewegung in leichter Sprache)<br>- Am Berufskolleg für Erzieher/-innen wurde ein Schwerpunkt zu „Gesundheit und Bewegung“ geschaffen.	- Schwierigkeiten bei der Mobilisierung von Teilnehmer/-innen (zu kurzfristig)<br>- Herforder Eltern, Erzieher/-innen (und Lehrer/-innen) wurden nachhaltig für das Thema „Essen und Bewegung“ sensibilisiert - einzelne Projekte wurden zum Teil fortgeführt<br>- Beratung durch Hebammen und Kinderärzt/-innen wird weiterhin angeboten und nachgefragt<br>- Erfolg auch durch regelmäßige Berichterstattung der lokalen Presse		Quelle: http://www.kidsvital.de/die-kids-vital-projekte/p6-familien-aktionen
Muttersprachliche Eltern-begleiter*innen für Kindergesundheit	Seit 2007	Gesundheitsamt Dortmund	- Ausbildung von Elternbegleiter/-innen in 12 Treffen mit folgenden Bausteinen der Qualifizierung:<br>a) Überblick über das deutsche Gesundheitssystem<br>b) gesunde Familienernährung, Bewegung und Entspannung im<br>c) Kindergesundheit und Kinderkrankheiten (Vorsorgeuntersuchungen und Impfungen)<br>d) Umgang mit Medien im frühen Kindesalter<br>e) Überblick über Hilfeangebote für Familien sowie Praxisübungen für eine erfolgreiche Elternarbeit		EP-A.: Ziel ist es, die Eltern so zu unterstützen, dass ihre Kompetenzen, die zur gesunden Entwicklung ihres Kindes beitragen, zu stärken	https://www.gesundheitliche-chancengleichheit.de/praxisdatenbank/muttersprachliche-elternbegleiter-innen-fuer-kindergesundheit/
Projekte – eingebettet in Präventionsketten						
BEAGEL		Stadt Arnsberg	- kostenlose Lotsenangebote für alle (werdenden) Eltern, z.B. durch „Familienlotsen“ oder „Schreibaby-Beratung“			https://beagle.nrw/das-projekt

			- Freiwilligkeit des Angebotes (Wunsch nach Unterstützung), ganzheitliche und gesundheitsorientierte Beratung, frühzeitige Unterstützung (z.B. vor oder bei Entstehung von Problemen, in benachteiligten oder veränderten Familiensituationen, erste Signale bei Überlastung, nicht günstigen Entwicklungsmöglichkeiten)<br>- Die Vermittlung erfolgt auf Empfehlung von anderen Eltern oder durch Fachkräfte wie z.B. niedergelassene und stationäre Fachärzte für Frauenärzt/-innen, freiberufliche Hebammen, Elternschule des Klinikum Hochsauerland, Schwangerschaftsberatungsstellen, Integrationsbüro, Jobcenter, Jugendamt, niedergelassene und stationäre Fachärzt/-innen für Kinder-und Jugendmedizin.<br>- individuellen Beratung verschiedene Themenbereiche wie z.B.: Ernährung, Bewegung, Mundgesundheit, Stillförderung – Stillzeit, Pflege, Hygiene, Sicherheit, Umgang, Handling, Unfallschutz, Bindungsförderung (Mutter-Kind /Vater-Kind), Sucht und Folgeschäden durch schädliche Substanzen, chronische Erkrankung, Behinderung, psychische Erkrankung, Stress, Entspannung, Entlastung<br>- zeitliche Ausrichtung ist hinsichtlich des Bedarfs in den jeweiligen Lebensphasen (Schwangerschaft, Geburt, erstes Lebensjahr) unterschiedlich			
Begrüßungsbesuche	Seit 2005	Gelsenkirchen – Team Familienförderung/-bildung	- Besuch alle Eltern zur Geburt ihres 1. Kindes (1.-8. Woche post partum)<br>- den Eltern wird eine Möglichkeit gegeben, Fragen und Anliegen persönlich zu besprechen und sich über die Familienbildungsangebote im nahen/eigenen Wohnumfeld zu informieren.<br>- persönliche Gespräch: Eltern bekommen die Gelegenheit, alle Themen anzusprechen, die sie beschäftigen – z.B. nach einem Betreuungsplatz, nach	- Durchgeführte Begrüßungsbesuche bis 31.10.2011: 5157 83,9% der Familien mit Erstgeborenen wurden erreicht (45,3% der Familien haben Migrationshintergrund)<br>- die Teilnahme an Elternbildungsangeboten in dem Stadtteil steigt seit der Einführung der Besuche deutlich		https://www.gelsenkirchen.de/de/_meta/aktuelles/artikel/anhang/439-pi0711_-_jedem_kind_seine_chance.pdf

			Krabbelgruppen oder der richtigen Säuglingskost.<br>- Aushändigung „Begrüßungspaket: „Gelsenkirchener Informationsbuch“, Elternbriefe, Broschüren, u.v.m.<br>- Der Besuch durch die Mitarbeiter/-innen des Familienbüros ist für Eltern freiwillig und kann kostenlos wahrgenommen werden.			
Café Kinderwagen		Kreis Warendorf als eine teilnehmende Kommune von Kinderstrak NRW schafft Chancen<br>- Initiator: Amt für Kinder, Jugendliche und Familien des Kreises Warendorf	- 14 Standorte mit jeweils einer Hebamme und einer pädagogischen Kraft<br>- das Konzept wird vom trägerübergreifenden, stadtweiten Netzwerk unterstützt und schafft so einen schnellen und unkomplizierten Zugang zu Hilfen und Unterstützungsangeboten<br>- Ziel: frühe Förderung der Kinder und der elterlichen Kompetenzen in einem entspannten Umfeld<br>- Enge Zusammenarbeit mit Familienzentrum und einer U3-Arbeitsgruppe des Netzwerkes „Frühe Hilfen und Schutz“<br>- Wiegekarte für jedes Kind, die an die Eltern beim ersten Besuch ausgegeben wird regelmäßige Gewichtskontrolle auf Wunsch der Eltern<br>- Hebamme nehmen eine erste Einschätzung zum Entwicklungsstand des Kindes vor Grundlage für Austausch über Kindliche Bedürfnisse	- Erfolgreich, da mehr als 10 verschiedene Kooperationspartner trägerübergreifend und zielgerichtet zusammenarbeiten<br>- großer Teil der Eltern im Kreis Warendorf werden erreicht; 37 Prozent aller Besucher/-innen nehmen kein anderes Angebot wahr besondere Ansprache der schwer erreichbaren Zielgruppe<br>- Sozial selektive Kriterien sind nicht erkennbar<br>- Knapp zwei Drittel aller Eltern sind durch die Hebamme auf das Angebot aufmerksam geworden	EP-A: Unterstützung durch pädagogische Fachkräfte	https://www.kinderstark.nrw/praxisbeispiele/detail?tx_news_pi1%5Baction%5D=detail&tx_news_pi1%5Bcontroller%5D=News&tx_news_pi1%5Bnews%5D=55&cHash=f7ce85cca6c2138c14f9283460b174bc
HAG		HAG in Hamburg	- Aus- und Fortbildung von pädagogischen Fachkräften aus der Kinder- und Jugendhilfe zu Ernährungsthemen und Beratung Elterngruppen in Einrichtungen.<br>- Fortbildungen für pädagogische Fachkräfte aus Eltern-Kind-Zentren, Elternschulen, Mütterberatungsstellen, Einrichtungen der Jugendhilfe und -bildung<br>- Ziel: Mitarbeiter/-innen der Einrichtungen aktuelle Empfehlungen zum Thema Ernährung von Säuglingen/Kleinkindern bzw. Jugendlichen zu geben, damit diese im Dialog mit Eltern bzw. Jugendlichen zu		Zu HAG:<br>- landesweites Kompetenzzentrum für Gesundheitsförderung in Hamburg<br>- verbindet Akteur/-innen aus den entscheidenden Sektoren und Arbeitsgebieten<br>- wird finanziert aus Mitteln der Behörde für Arbeit, Gesundheit, Soziales, Familie und	https://www.hag-gesundheit.de/fileadmin/hag/data/Arbeitsfelder/Gesund_aufwachsen/Familie/Ernaehrungspaedagogische_Angebote/Kurzbeschreibung_Module_Elternberatungen_2019.pdf

			diesem Thema auskunftsfähiger und sicherer sind.<br>*Themen der letzten Fortbildungen:*<br>„Ernährungspädagogik“:<br>- Wie lernen Kinder essen und wie können Eltern sie dabei gut begleiten?<br>- Vegane und vegetarische Ernährung?<br>- Antworten zu zwei besonderen Ernährungsformen, Körpergewicht – Welches ist richtig?		Integration (Sozialbehörde) sowie aus Projektmitteln von Krankenkassen (GKV), anderen Trägern und Mitgliedsbeiträgen	
Mo.Ki „unter 3“ Frühes Fördern von Anfang an	Seit 2005	Stadt Monheim am Rhein, AWO Bezirksverband Niederrhein e. V., begleitet durch Frankfurter ISS, finanzielle Förderung Stiftung Wohlfahrtspflege und Landesverband Rheinland	- zahlreiche Angebote für Eltern von der Schwangerschaft bis zur Vollendung des dritten Lebensjahres mit den Schwerpunkten<br>a) „Begrüßungsbesuche“:<br>- Familien mit neugeborenen Kindern oder zugezogene Familien mit Kindern unter drei Jahren werden von Mitarbeiter/-innen der Familienhilfe besucht und erhalten Begrüßungsordner mit Infomaterialien und Gutscheinen<br>- Erfassung von Bedürfnissen und Wünschen der kontaktierten Familie<br>- Aufklärung und Vermittlung zu primär- und sekundärpräventiven Angeboten wie Erstberatung des Jugendamtes und Sprechstunde der Familienhilfe<br>Motivation zur Kontaktaufnahme und zum Besuch der Mo.Ki-Cafés<br>Bei Bedarf Vermittlung zu Ämtern, Behörden, Diensten<br>b) „Mo.Ki. Cafés“:<br>- zwei Mo.Ki-Cafés<br>- Organisation und Durchführung eines täglichen Frühstückangebots<br>- Anlaufstelle für Familien für alle Lebensfragen und in akuten Notlagen<br>- Infobörse zu allen Fragestellungen rund um das Thema Geburt, Erziehung und Gesundheit<br>Konkrete Hilfestellungen, z.B. Online-Anmeldung zum Kindergarten<br>Verbindungen schaffen zwischen Gästen und Kooperationspartnern<br>Durchführung niederschwelliger und alltagsorientierter Bildungsangebote	- Zugangs- und Nutzungschancen vor allem für benachteiligte Gruppen durch breit gefächertes Angebot erhöht		https://www.monheim.de/kinder-jugend/unterstuetzung-fuer-familien-von-anfang-an

			c) „Familienbildung“:<br>- Breites Spektrum an Veranstaltungen und Kursen, das sich am Bedarf junger Familien orientiert<br>d) „Familienhebammen“:<br>- Zwei Familienhebammen begleiten in Form regelmäßiger Hausbesuche und Sprechstunden die Familien bis zum vollendeten ersten Lebensjahr, die gesundheitlichen und psychologischen Risiken ausgesetzt sind<br>*Kursangebote rund um die Geburt:*<br>- Hebammensprechstunde<br>- Geburtsvorbereitung<br>- Rückbildungsgymnastik<br>- Yoga für Schwangere<br>*Kursangebote bis zum dritten Lebensjahr:*<br>- Angebote für Eltern: Strickkreis, Mo.Ki-Elternkurs (Erfahrungsaustausch und Diskussion verschiedener Erziehungsthemen), Marte-Meo-Elternkurs (gegenseitiges Kennenlernen von Eltern und Kind)<br>- Angebote im Jahresverlauf: Gesundheits- und Ernährungswochen, Ferienprogramm in den Sommerferien, Sprach- und Lesewoche (Sprachentwicklung und -förderung), Fete und Feiern (z.B. Eiersuchen an Ostern, Überraschungen zum islamischen Opferfest u.v.m.), Weihnachtswerkstatt (Herstellen von Weihnachtsgeschenken und -dekorationen			
