# Supplementary material for: Community-based care structures to promote nutritional health in the first 1000 days—Analysis of the status quo
Source: Pravent Gesundh. 2022 Nov 28:1–15. [Article in German] Online ahead of print. doi: 10.1007/s11553-022-00998-2 (PMC9707151; doi:10.1007/s11553-022-00998-2)
Supplement: Supplementary file 2 — Tabelle S2 Ausgeschlossene (kommunal verankerte) Projekte [file 11553_2022_998_MOESM2_ESM.pdf]

Tabelle S2 Ausgeschlossene (kommunal verankerte) Projekte

**Titel**	**Träger**	**Grund für Ausschluss**
AGIL - Aktiver Gesünder Is(s)t Leichter	Kinderschutzbund Aachen	- richtet sich an Kinder von 4-10 Jahren
Fit im Leben – mit Spaß und Karla dabei!	Verbandsgemeinde Hillesheim/Vulkaneifel	- richtet sich hauptsächlich an ältere Kinder
Gesundheit jetzt – in sozialen Brennpunkten	Armut und Gesundheit in Deutschland e.V.	- nicht primäre Zielgruppe
Kinderleicht-Quartier - stärken. Was wirkt!	Verein Gesundheitshaus Gelsenkirchen e.V.	- richtet sich eher an ältere Kinder
Macht euch auf die Socken	Kreis Herford - Der Landrat – Gesundheit	- richtet sich eher an ältere Kinder
Medizinische Versorgung und gesundheitsfördernde Angebote in der Gütersloher Suppenküche und der Kinderküche "die Insel"	Landeszentrum Gesundheit Nordrhein-Westfalen	- Kein Präventionsprogramm
optiSTART	Stadt Leipzig, Gesundheitsamt	- nicht primäre Zielgruppe<br>- richtet sich eher an ältere Kinder
TigerKids - Kindergarten aktiv	Stiftung Kindergesundheit	- nicht primäre Zielgruppe<br>- richtet sich eher an ältere Kinder
